# Dopaminergic Stimulation of Myeloid Antigen-Presenting Cells Attenuates Signal Transducer and Activator of Transcription 3-Activation Favouring the Development of Experimental Autoimmune Encephalomyelitis

**DOI:** 10.3389/fimmu.2018.00571

**Published:** 2018-03-21

**Authors:** Carolina Prado, Michela Gaiazzi, Hugo González, Valentina Ugalde, Alicia Figueroa, Francisco J. Osorio-Barrios, Ernesto López, Alvaro Lladser, Emanuela Rasini, Franca Marino, Mauro Zaffaroni, Marco Cosentino, Rodrigo Pacheco

**Affiliations:** ^1^Laboratorio de Neuroinmunología, Fundación Ciencia and Vida, Santiago, Chile; ^2^Center for Research in Medical Pharmacology, University of Insubria, Varese, Italy; ^3^Laboratorio de Inmunoterapia Génica, Fundación Ciencia and Vida, Santiago, Chile; ^4^Multiple Sclerosis Centre, ASST della Valle Olona, Hospital of Gallarate, Gallarate, Italy; ^5^Departamento de Ciencias Biológicas, Facultad de Ciencias Biológicas, Universidad Andres Bello, Santiago, Chile

**Keywords:** multiple sclerosis, experimental autoimmune encephalomyelitis, antigen-presenting cells, CD4^+^ T cells, dopamine

## Abstract

The dual potential to promote tolerance or inflammation to self-antigens makes dendritic cells (DCs) fundamental players in autoimmunity. Previous results have shown that stimulation of dopamine receptor D5 (DRD5) in DCs potentiates their inflammatory behaviour, favouring the development of experimental autoimmune encephalomyelitis (EAE). Here, we aimed to decipher the underlying mechanism and to test its relevance in multiple sclerosis (MS) patients. Our data shows that DRD5-deficiency confined to DCs in EAE mice resulted in reduced frequencies of CD4^+^ T-cell subsets with inflammatory potential in the central nervous system, including not only Th1 and Th17 cells but also granulocyte-macrophage colony-stimulating factor producers. Importantly, *ex vivo* depletion of dopamine from DCs resulted in a dramatic reduction of EAE severity, highlighting the relevance of an autocrine loop promoting inflammation *in vivo*. Mechanistic analyses indicated that DRD5-signalling in both mouse DCs and human monocytes involves the attenuation of signal transducer and activator of transcription 3-activation, a transcription factor that limits the production of the inflammatory cytokines interleukin (IL)-12 and IL-23. Furthermore, we found an exacerbated expression of all dopamine receptors in peripheral blood pro-inflammatory monocytes obtained from MS patients. These findings illustrate a novel mechanism by which myeloid antigen-presenting cells may trigger the onset of their inflammatory behaviour promoting the development of autoimmunity.

## Introduction

T-cell mediated immunity can respond to a huge variety of foreign and self-antigens as a result of a wide repertoire of T-cell receptors generated by genomic recombination. To avoid autoimmune responses, self-reactive T-cells have to be eliminated or rendered tolerant, a process in which dendritic cells (DCs) play a fundamental role. Indeed, constitutive depletion of DCs leads to spontaneous fatal autoimmunity (1). On the other hand, during onset of autoimmunity DCs seem to be critical for priming of self-reactive T-cells that escape of tolerance induction (2). This dual potential of DCs promoting tolerance or inducing inflammation to self-antigens makes these cells fundamental players in the physiopathology of autoimmunity.

Unravelling molecular mechanisms controlling the switch between the tolerogenic and the pro-inflammatory behaviours of DCs results of pivotal importance to understand the development of autoimmune disorders. In this regard, a master inducer of the pro-inflammatory potential of DCs is the transcription factor nuclear factor kappa B (NF-κB), which may be activated by the integration of many different inflammatory cues, including pro-inflammatory cytokines, pattern-recognition receptors stimulation, and the inflammasome (3–5). Importantly, NF-κB activation in DCs has been associated with the production of interleukin (IL)-12 and IL-23, which are, respectively, involved in the induction of Th1 and Th17 immunity (6, 7). Conversely, by inducing the expression of anti-inflammatory mediators, such as IL-10, TGF-β, and retinaldehyde dehydrogenase, the activation of β-catenin has been described to play a pivotal role in the acquisition of the tolerogenic potential by DCs (8, 9). Another key regulator of DCs behaviour is the transcription factor signal transducer and activator of transcription 3 (STAT3), which upon phosphorylation inhibits the NF-κB-mediated induction of IL-12 and IL-23, attenuating the inflammatory potential of these cells (10, 11).

Previous evidence suggested that Th1 as well as Th17 cells are the phenotypes of autoreactive T-cells involved in autoimmunity, including multiple sclerosis (MS) and its mouse model, the experimental autoimmune encephalomyelitis (EAE) (12, 13). Despite there are several studies indicating that Th17 and Th1 cells infiltrate the central nervous system (CNS) during the course of EAE development, more recent studies have shown that mice bearing IL-17- or interferon (IFN)-γ-deficient CD4^+^ T-cells, still developed EAE, although with decreased severity. In this regard, irrespective of the secretion of IL-17 and IFN-γ, the production of granulocyte-macrophage colony-stimulating factor (GM-CSF) by autoreactive CD4^+^ T-cells was essential for the development of EAE (14, 15). Thus, the pro-inflammatory cytokines IL-17, IFN-γ, and GM-CSF produced by CD4^+^ T-cells contribute to the development of EAE, although the latter plays a major role (14, 16). The production of this cytokine is induced in CD4^+^ T-cells by the concerted action of IL-23 and IL-12 (14, 15, 17). Thereby DCs, by means of the secretion of IL-12 and IL-23 could regulate the production of GM-CSF by CD4^+^ T-cells. On the other hand, CD4^+^ regulatory T-cells (Tregs) infiltrate progressively into the CNS starting on the disease onset. However, the generation and the suppressive activity of CNS-infiltrated Tregs are impaired by γδT-cells (18). This latter T-cell subset infiltrates the CNS during EAE, starting at the disease onset, reaching the highest cell number at the peak of disease manifestation, and then progressively disappearing from the CNS, thus correlating with the time-course of EAE manifestation (18). γδT-cells inhibit CD4^+^ Tregs function and produce massive amounts of IL-17 in response to IL-23. Once γδT-cells begin disappearing from CNS, CD4^+^ Tregs continue progressively infiltrating CNS and they recover their suppressive activity, which correlates with progressive attenuation of EAE manifestation (18). In addition, there is a number of studies pointing to the participation of a regulatory subpopulation of CD8^+^ T-cells which dampen EAE severity (19, 20). In this regard, distinct subpopulations of CD8^+^ Tregs have been described, including CD8^+^CD122^+^, CD8^+^CD28^−^, and CD8^+^LAP^+^ (21–23). Altogether these findings strongly suggest that DCs, by regulating the activation and differentiation of both CD4^+^ and CD8^+^ T-cells, play a fundamental and complex role in the development of T-cell response involved in EAE. Regarding the pro-inflammatory function of antigen-presenting cells (APCs) in human, peripheral blood monocytes have been shown to represent an important source of inflammatory cytokines, including IL-12 and IL-23 (24, 25). Furthermore, it has been shown that the production of inflammatory cytokines is exacerbated in peripheral blood monocytes obtained from MS patients (25). Thus, these findings suggest that, in addition to DCs, human peripheral blood monocytes may also play a relevant immunomodulatory role as APCs in the physiopathology of MS.

Among five dopamine receptors (DRs) described so far, D_1_-like DRs [including dopamine receptor D1 (DRD1) and DRD5] as well as D_2_-like DRs (including DRD2, DRD3, and DRD4) have been found in immune cells (26, 27). Emerging evidence suggests a relevant role for the dopaminergic regulation in the control of MS and EAE development (27–31). During EAE onset and at the peak of disease manifestations, there is an important increase of dopamine levels in the striatum (32). In addition, a reduced expression of DRD5 has been described in peripheral blood mononuclear cells (PBMCs) obtained from untreated MS patients when compared with PBMCs obtained from healthy donors (33). Furthermore, when patients were treated with IFN-β, a cytokine that attenuated disease manifestation, a progressive increase on DRD5 expression was observed in PBMCs during the treatment period (34). Further studies analysing DRs in different T-cell subsets revealed an opposite deregulation of DRD5 expression in Tregs and Teffs obtained from MS patients (35). Importantly, these studies strongly suggest the involvement of DRD5-signalling in immune cells in the physiopathology of MS.

Of note, previous findings have demonstrated that DCs, from human or mouse origin, express the whole machinery to synthesise and store dopamine, which can be released upon maturation or antigen presentation (36, 37). Moreover, *in vitro* analyses have indicated that DRD5-stimulation in DCs favours the production of IL-12 and IL-23 by DCs (37). Since IL-12 and IL-23 can stimulate, respectively, Th1 and Th17 differentiation and also promote γδT-cell function (18) and generation of GM-CSF-producing CD4^+^ T-cells (14), all of them representing cell subpopulations involved in EAE pathogenesis, it is likely that DRD5-signalling in DCs plays a relevant role in EAE.

In this study, we aimed to unravel the relevance of DRD5-signalling in DCs in the development of EAE and the underlying mechanism involved in the induction of an autoreactive T-cell mediated response. Moreover, we also studied the association of DRD5-signalling in APCs with MS. Our findings show here that DRD5-signalling confined to DCs promotes selectively the participation of inflammatory T-cell subsets in the CNS of EAE mice, including Th1, Th17, and GM-CSF-producing CD4^+^ T-cells, without affecting suppressive T-cells. Mechanistic analyses demonstrated that DRD5-signalling was triggered by an autocrine loop exerted by dopamine derived from DCs. Furthermore, our results indicate that DRD5-signalling in DCs was mediated through the attenuation of STAT3-activation. Finally, our data obtained from human individuals indicates that DRD5–STAT3 axis is also present in human monocytes and this signalling is upregulated in MS patients.

## Materials and Methods

### Animals

Six- to eight-week-old mice of the C57BL/6 background were used for all experiments. Wild-type (WT) C57BL/6 mice were purchased from The Jackson Laboratory (Bar Harbor, ME, USA). DRD5-knockout (DRD5KO) mice were kindly donated by Dr. David Sibley (38), which were backcrossed for at least 10-generations in the C57BL/6 genetic background. CD11c.DOG mice in the C57BL/6 genetic background were kindly donated by Dr. Natalio Garbi and Günter Hämmerling (39). All mice were maintained and manipulated according to institutional guidelines at the pathogen-free facility of the Fundación Ciencia & Vida.

### Reagents

PerCP–anti-CD4 (GK1.5), PerCP–anti CD8 (53-6.7), allophycocyanin–anti-IFN-g (XMG1.2), phycoerythrin (PE)-Cy7–anti-IL-17A (TC11-18H10.1), PE–anti-GM-CSF (MP1-22E9), Brilliant Violet 421–anti-TCRγδ (GL3), Brilliant Violet 421–anti-leucocyte alkaline phosphatase (LAP) (TW7-16B4), PE–anti-CD122 (5H4), allophycocyanin–anti CD28 (E18) Abs, and Cell Trace Violet were purchased from BioLegend. Allophycocyanin–anti-Foxp3 (FJK16s) antibody (Ab) was obtained from eBioscience. Anti-phospho-STAT3 (3E2) and anti-STAT3 (79D7) were obtained from Cell Signaling. Phorbol 12-myristate 13-acetate (PMA) and ionomycin were purchased from Sigma-Aldrich. Horseradish peroxidase (HRP)–anti-rabbit IgG Ab was obtained from Santa Cruz Biotechnology. HRP-conjugated anti-mouse IgG Ab was purchased from Rockland Immunochemicals. Brefeldin A was obtained from Life Technologies. The pMOG (MOG_35–55_) was purchased from GeneTel Laboratories (Madison, WI, USA).

### Generation of Mouse DCs

Bone marrow-derived DCs (BM-DCs) from WT and DRD5KO mice were prepared as previously described (37). Briefly, DCs were grown in RPMI 1640 medium (Hyclone, Logan, UT, USA) supplemented with 10% heat-inactivated FBS (Biological Industries, Beit Haemek, Israel) and 10 ng/ml recombinant mouse GM-CSF (PeproTech, Rocky Hill, NJ, USA). On day 5, differentiation of DCs was routinely assessed obtaining <80% CD11c^+^ cells. In some experiments, day 5 DCs were either left unstimulated or stimulated with 100 ng/ml lipopolysaccharide (LPS) (Sigma Chemical Co., St. Louis, MO, USA) for 24 h and used for further experiments.

### Intracellular Cytokine Staining Analysis

To analyse cytokine production, cells were restimulated with 1 mg/ml ionomycin and 50 ng/ml PMA for 4 h in the presence of 5 mg/ml brefeldin A. For intracellular staining, cells were first stained with a Zombie Aqua fixable viability kit (BioLegend), followed by staining for cell surface markers. Intracellular staining was done with the Foxp3 staining buffer set (eBioscience). Data were collected with a FACSCanto (BD Biosciences) and analysed with FlowJo software (Tree Star).

### Western Blot

To analyse the phosphorylation of STAT3, 2 × 10^6^ cells/ml DCs were cultured in the presence or the absence of 100 ng/ml LPS and either left untreated or treated with 1 µM SCH23390 (SCH) or 1 µM reserpine (RSP; Tocris, Bristol, UK) for 8 h. Samples containing 50 µg protein were run on denaturing conditions and transferred to polyvinylidene difluoride membranes (Thermo Scientific). Phosphorylated STAT3 (pSTAT3) was detected with a mouse monoclonal antibody and HRP-conjugated secondary Ab against mouse IgG. STAT3 expression was detected using a rabbit polyclonal Ab and HRP-conjugated secondary Ab against rabbit IgG. Immunodetection was carried out using SuperSignal West Femto chemiluminescent substrate (Thermo Scientific).

### Lentiviral Transduction

Constitutively active STAT3 (STAT3ca, Cys substitutions at Ala662 and Asn664) cDNA were cloned in pLNCX vector and kindly provided by Dr. Robert Arceci (40). For the construction of the lentiviral vector codifying STAT3ca, mouse STAT3ca sequence was excised from pLNCX vector and subcloned into a pLVX-IRES-ZsGreen1 vector (Clonetech). Recombinant lentiviruses were produced by transfecting HEK293T with the lentiviral expression plasmid pLVX-IRES-ZsGreen1 or pLVX-STAT3ca and the packaging plasmids pCMV-dR8.91 and pCMV-VSV-G using Turbofect (Termofisher) according manufacturer instructions. Infectious lentiviruses were harvested at 48 h post-transfection and then concentrated. The infectious titre was determined by FACS by quantifying expression of green fluorescent protein (GFP) in infected HEK293T cells. DCs were transduced with STAT3ca or control lentivirus on days 4 and 5 of differentiation at MOI 10 in the presence of 2 µg/ml polybrene.

### EAE Induction and Evaluation

Experimental autoimmune encephalomyelitis induction and the transfer of DCs were carried out as described before (41). Briefly, 6- to 8-week-old female WT, DRD5KO, or CD11c.DOG mice were injected s.c. with 50 mg pMOG (Genetel Laboratories, Madison, WI, USA) emulsified in complete Freund’s adjuvant (CFA) (Invitrogen) supplemented with heat-inactivated *Mycobacterium tuberculosis* H37 RA (Difco Laboratories, Detroit, MI, USA). In addition, mice received i.p. injections of 500 ng pertussis toxin (Calbiochem, La Jolla, CA, USA) on days 0 and 2. Clinical signs were assessed daily according to the following scoring criteria: 0, no detectable signs; 1, flaccid tail; 2, hind limb weakness or abnormal gait; 3, complete hind limb paralysis; 4, paralysis of fore and hind limbs; and 5, moribund or death. In some EAE experiments, 10^6^ BM-DCs from WT and DRD5KO mice were pulsed with 5 mg/ml pMOG for 4 h and then i.v. transference were done into WT C57BL/6 recipient mice 14 and 7 days before EAE induction. In experiments using CD11c.DOG mice as recipients, animals were daily injected with Diphteria Toxin (8 μg/Kg) to eliminate endogenous DCs (39) from day 9 to day 5 before EAE induction. These mice received the i.v. transference of pMOG-pulsed WT or DRD5KO DCs on day 7 before EAE induction. For the preparation of CNS mononuclear cells, mice were transcardially perfused with cold PBS. The brain and spinal cord were dissected, and CNS tissue was cut into small pieces and digested by collagenase D (2.5 mg/ml; Roche Diagnostics) and DNaseI (1 mg/ml; Sigma) at 37°C for 45 min. Digested tissue was passed through a 70-mm cell strainer obtaining single cell suspension that was subjected to centrifugation in a Percoll gradient (70/40%). Mononuclear cells were removed from the interphase and resuspended in culture medium for further analysis.

### Evaluation of cAMP and Dopamine

Dendritic cells were harvested, washed, and resuspended in RPMI 1640 medium at 10^6^ cells/ml. cAMP was determined as described (42). Briefly, cells were incubated with 50 nM of Zardaverine (Tocris) for 30 min at 37°C and then treated with or without dopamine (Sigma) at indicated concentrations in the presence or absence of 500 nM Forskolin (Tocris). After incubating for 45 min at 37°C, cells were lysed and cAMP concentration was determined using cAMP-Screen Direct System (Applied Biosystems) according to the manufacturer’s instructions. To determine the levels of dopamine released into the culture supernatant, DCs were treated with the indicated stimuli at 37°C for 60 min and then the supernatant was separated by centrifugation. Dopamine levels were quantified in the supernatant by using an ELISA-based kit (Alpco Diagnostics) following the manufacturers recommendations.

### Human Subjects

Patients with relapsing/remitting MS according to the revised McDonald’s criteria (43) treated with IFN treatment according to the Italian Ministry of Health’s guidelines were included in the study (see Table 1). Patients were enrolled at the MS Center of the Hospital S. Antonio Abate, Gallarate (VA, Italy). Healthy subjects (HS) were usually spouses and caregivers of enrolled MS patients. Subjects with neuropsychiatric disorders (e.g., depression) were excluded from the study. At the time of enrolment, all patients were free from relapse since at least 1 month, no one was receiving sympathoadrenergic agents, dopaminergic agents, or antidepressants at least during the prior 3 months nor steroid treatment in the last month. Functional disability was measured using the Kurtzke expanded disability status score (44).

**Table 1:** Characteristics of the subjects included in the study.

	Healthy subjects	Multiple sclerosis patients	*p*
*n* (F/M)	10 (7/3)	9 (5/4)	0.650
Age (years)	42.3 ± 8.4	46.4 ± 9.5	0.326
Disease duration (years)		19.4 ± 9.3	
Baseline expanded disability status score (median ± SD)		2.9 ± 2.1	
**Treatments^a^**			
Interferon (IFN)-β1a			
– Avonex^®^, Biogen		2	
– Plegridy^®^, Biogen		2	
– Rebif44^®^, Serono		1	
**IFN-β1b**			
– Extavia^®^, Novartis		3	
– Betaferon^®^, Bayer		1	

*Values are mean ± SD unless otherwise indicated*.

*^a^According to the approved therapeutic regimens*.

Venous blood samples were obtained after a fasting night, between 8:00 a.m. and 10:00 a.m., in universal tubes containing 10.7 mg ethylenediaminetetraacetic acid dipotassium salt. Tubes were subsequently coded and stored at room temperature (RT) until processing, which occurred within 2 h after collection.

### Flow Cytometric Analysis of Human DCs and Monocytes and of DRs Expression

Analysis of DRs on DCs and monocytes was performed according to a previously established method (45) with modifications. Briefly, 200 μl aliquots of whole blood were prepared and erythrocytes were removed by means of a lysis buffer [composition (g/l in ultrapure water): NH_4_Cl 8.248, KHCO_3_ 1.0, EDTA 0.0368]. Incubation was performed at RT for 5 min, during which samples were gently vortexed. Samples were then centrifuged at 600 *g* for 5 min at RT, supernatants were removed, and cells were washed one time in 1 ml of PBS [composition (g/l in ultrapure water): NaCl 8.4, Na_2_HPO_4_ 1.424, NaH_2_PO_4_ 0.276] at pH 7.4 supplemented with 1% BSA (Sigma, Italy) (PBS/BSA), once more centrifuged at 600 *g* for 5 min at RT, and finally resuspended in 100 µl PBS/BSA. From each subject, 13 aliquots of whole blood were prepared and processed for evaluation of DCs (6 aliquots: 5 for DRs staining, 1 as control for the secondary Ab) and of monocytes (7 aliquots: 5 for DRs staining, 1 as control for the secondary Ab, and 1 for the anti-CD16 Ab isotype control).

The staining protocol consisted of two steps. During the first step, each aliquot was treated with 5 µl Fc block solution for 10 min at RT to prevent any unwanted binding of anti-human Ab to Fc receptors and thereafter stained for one of the five DRs by an indirect labelling procedure (primary Ab + secondary Ab). Aliquots were incubated with the primary anti-DR Ab for 30 min on ice in the dark, washed one time with PBS/BSA at 600 g for 5 min at RT, and resuspended in 100 µl PBS/BSA containing the secondary Ab and incubated for 30 min on ice in the dark. Aliquots were then washed (600 g for 5 min at RT) and once more resuspended in 100 µl PBS/BSA. During the second step, the aliquots for evaluation of DCs were incubated with a cocktail of anti-human Lin1, human leucocyte antigen DR (HLA-DR), CD123, and CD11c, for the identification of total circulating DCs (Lin1-/HLA-DR^+^), plasmacytoid DCs (pDCs, CD123^high^/CD11c^−^), and myeloid DC (mDC, CD123^low^/CD11c^high^), whilst the aliquots for evaluation of monocytes were incubated with a cocktail of anti-human CD45, HLA-DR, CD14, and CD16 for the identification of classical (CD14^high^/CD16^−^), non-classical (CD14^low^/CD16^high^), and intermediate (CD14^high^/CD16^+^) monocytes. As a control of isotype, cells were stained with an IgG1k of irrelevant specificity instead of the anti-human CD16 Ab. All aliquots were incubated for 20 min in dark at RT, washed with 1 ml of PBS/BSA (600 g for 5 min at RT), finally resuspended in 600 µl PBS and kept on ice until analysis. The complete list of Ab used in the protocols together with their working dilutions is shown in Table S1 in Supplementary Material.

Acquisition and analysis were performed on a BD FACSCanto II flow cytometer (Becton Dickinson, Milan, Italy) with BD FACSDiva software (version 6.1.3). The gating strategies used to identify DCs and monocytes are shown in Figures S9 and S10 in Supplementary Material, respectively. The results were finally expressed as absolute numbers (10^3^/mm^3^) as well as percentage of positive cells (%), as well as mean fluorescence intensity (MFI) of positive cells, calculated as the difference between MFI in anti-human DR Ab stained aliquots and aliquots stained with the secondary Ab alone.

### Flow Cytometric Assay of pSTAT3 in Human Monocytes

Samples of 200 µl whole blood from HS were kept for 5 min on ice in the dark, alone or in the presence of different concentrations of dopamine hydrochloride (Sigma, Italy; code H8502). Thereafter, samples were added with anti-human CD14 Ab and incubated for 20 min at 37°C in a water bath. During this period, 100 ng/ml IL-6 (Biolegend, San Diego, CA, USA; code 570804) was eventually added after 5 min.

Erythrocyte lysis and cell fixation and permeabilisation for intracellular staining of pSTAT3 were then performed according to BD Phosflow Protocol III for Human Whole Blood (http://www.bdbiosciences.com/us/applications/research/intracellular-flow/m/745716/resources). Briefly, samples were added with 3 ml of pre-warmed Lys/Fix Buffer 1X and incubated for 12 min at 37°C in a water bath. Samples were then centrifuged at 600 *g* for 7 min at RT, supernatants were removed and cells were washed with 3 ml of Dulbecco’s PBS 1X [composition (g/l in ultrapure water): CaCl_2_ 0.1, MgCl_2_·6 H_2_O 0.1, KCl 0.2, KH_2_PO_4_ 0.2, NaCl 8.0, Na_2_HPO_4_·7 H_2_O 2.16] at pH 7.4. After another centrifugation (600 *g* for 7 min) supernatants were removed and cells were added with 1 ml of pre-chilled Perm Buffer III and incubated for 30 min on ice. Cells were then washed twice in 3 ml of Stain Buffer (600 g for 7 min each). Supernatants were thereafter removed, cells were resuspended in 100 µl of Stain Buffer and added with anti-human pSTAT3 ab. Samples were subsequently incubated for 30 min in the dark at RT, washed with 3 ml of stain buffer (600 g for 7 min), finally resuspended in 500 µl stain buffer and kept on ice until analysis. The complete list of Ab used in the protocol together with their working dilutions is shown in Table S1 in Supplementary Material.

Acquisition and analysis were performed on a BD FACSCanto II flow cytometer (Becton Dickinson, Milan, Italy) with BD FACSDiva software (version 6.1.3). The gating strategies used to identify pSTAT3^+^ monocytes are shown in Figure S7 in Supplementary Material. The results were finally expressed as percentage of positive cells (%), as well as MFI of positive cells, calculated as the difference between MFI in anti-human DR Ab stained aliquots and aliquots stained with the secondary Ab alone.

### Statistical Analysis

All values were expressed as mean ± SEM. Differences in means between two groups were analysed by two-tailed Student’s *t*-test. Progression of EAE severity curves were compared with a non-parametric Mann–Whitney rank sums two-tailed *U*-test. *P* value ≤0.05 was considered significant. Analyses were performed with GraphPad Prism 6 software.

### Study Approval

The study performed with human individuals conforms to the principles outlined in the Declaration of Helsinki, the study protocol was approved by the local Ethics Committee of the Ospedale di Circolo and Fondazione Macchi, Varese (Italy), and all the participants signed a written informed consent before enrolment. All procedures performed in animals were approved by and complied with regulations of the Institutional Animal Care and Use Committee at Fundación Ciencia & Vida.

## Results

### DRD5-Signalling in DCs Favours the Development of Aggressive EAE

**Figure 1:**
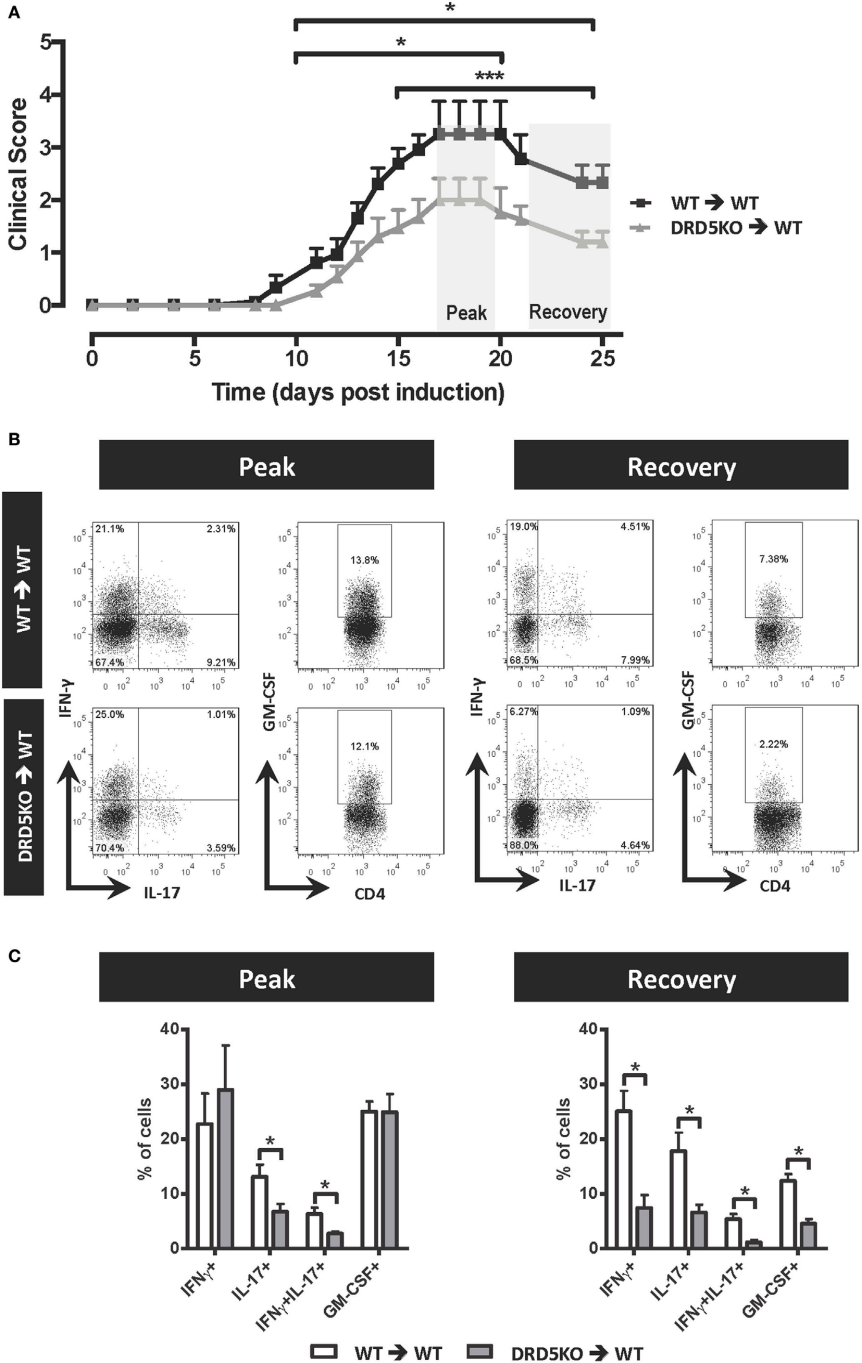
Lack of DRD5-signalling in dendritic cells (DCs) results in a reduction in the frequencies of Th1, Th17, GM-CSF^+^, and interferon (IFN)-γ^+^ IL17^+^ CD4^+^ T-cells infiltrating the central nervous system of experimental autoimmune encephalomyelitis (EAE) mice. Wild type (WT) (black lines) or DRD5-knockout (DRD5KO) (grey line) bone marrow-derived DCs were pulsed with pMOG and transferred (10^6^ DCs/mice; i.v. injections) into WT recipient mice at days 14 and 7 prior EAE induction. **(A)** Disease severity was evaluated as clinical score (see Materials and Methods) from day 0 to day 25 post-induction. Data from at least 14 mice per group are shown, corresponding to a representative from three independent experiments. Values represent mean ± SEM. *p < 0.05; ***p < 0.001 by Mann–Whitney *U*-test in the indicated time frames. **(B,C)** At the peak (17–19 dpi) or at the recovery phase (25 dpi) of the disease, mononuclear cells were isolated from central nervous system (CNS) followed by *ex vivo* stimulation with phorbol 12-myristate 13-acetate/ionomycin in the presence of brefeldin A, and intracellular cytokine staining analysis was carried out by flow cytometry. Inflammatory populations were analysed at the peak (left panels) or at the recovery phase (right panels). **(B)** Representative dot-plots for interleukin (IL)-17, IFN-γ, and granulocyte-macrophage colony-stimulating factor (GM-CSF) production in the infiltrating CD4^+^ gated population are shown. Numbers on the dot-plots indicate the percentage of cells in the associated region. **(C)** Frequencies of inflammatory T-cells infiltrating the CNS are represented as the mean ± SEM. *p < 0.05 by unpaired two-tailed Student’s *t*-test.

To determine whether DRD5 expressed in DCs was functional, we first tested the signalling pathway coupled to this receptor. Similar to most reports analysing the coupling of DRD5 in different cell types (46), our results show here that DRD5-stimulation in DCs was coupled with cAMP production (Figure S1 in Supplementary Material). To evaluate the role of DRD5-signalling confined to DCs in the development of EAE, we first performed a set of adoptive transfer experiments in which WT or DRD5KO DCs were loaded *ex vivo* with the autoantigen associated with EAE, a peptide derived from MOG (pMOG), and then transferred into WT recipients in which EAE was induced. The results show that DRD5-deficiency in DCs results in a significant attenuation in the severity of EAE manifestation during the peak of the disease manifestation and also during the recovery phase (Figure 1A). The experimental settings used in these experiments open the possibility that a fraction of pMOG-loaded DRD5KO DCs transferred were dying and phagocytosed by endogenous WT DCs, thus dampening the final effect of DRD5-deficiency in EAE. To rule out this possibility, we performed similar experiments but transferring pMOG-loaded DCs into WT recipients previously depleted of endogenous DCs. For this purpose, we used CD11c.DOG mice as recipients, which express the diphtheria toxin (DT) receptor under the control of CD11c-promoter (39). We depleted endogenous DCs in CD11c.DOG mice with DT during the time frame in which pMOG-loaded WT or DRD5KO DCs were transferred and then EAE development was analysed. These results show that lack of DRD5-signalling in pMOG-loaded DCs transferred into DCs-depleted recipient mice results in a significant reduction of EAE manifestation (Figure S2 in Supplementary Material). The reduction in EAE severity due to DRD5-deficiency in DCs was similar when endogenous DCs were depleted or not (Figure 1A; Figure S2 in Supplementary Material), thereby indicating that endogenous DCs did not interfere with the priming of pMOG-response induced by transferred pMOG-loaded *ex vivo* DCs.

### DRD5-Deficiency Confined to DCs Results in Reduced Frequencies of Inflammatory CD4^+^ T-Cells in the CNS of EAE Mice

In a previous study, we observed that DRD5-signalling in DCs was required to induce high frequencies of Th17 cells infiltrating the CNS during the peak of disease manifestation in EAE mice (37). To gain a wide and deeper insight in the mechanism involved in the pro-inflammatory effect of DRD5-signalling in DCs favouring the development of EAE, in this study, we analysed how it was affecting a broad panel of inflammatory and suppressive subsets of T-cell infiltrating the CNS during the peak of disease manifestation and during the recovery phase of EAE. For this purpose, we analysed the relative frequencies not only of Th1 and Th17 cells but also double positive IFN-γ^+^ IL-17^+^, and the highly inflammatory GM-CSF^+^ subsets including IFN-γ^+^ GM-CSF^+^, IL-17^+^ GM-CSF^+^, and IFN-γ^+^ IL-17^+^ GM-CSF^+^ CD4^+^ T-cells (14). The results show that despite DRD5-deficiency in DCs affected only Th17, IFN-γ^+^ IL-17^+^, and IFN-γ^+^ IL-17^+^ GM-CSF^+^ subsets of CD4^+^ T-cells during the peak of disease manifestation, Th1 and the highly inflammatory GM-CSF^+^ CD4^+^ T-cells were also significantly reduced in the CNS during the recovery phase of EAE (Figures 1B,C; Figure S3 in Supplementary Material). In addition, we evaluated the frequency of γδT-cells, which have been described to produce IL-17 and to inhibit the suppressive activity of Tregs in the CNS during the peak of EAE manifestation (18). However, we did not find differences in the extent of total γδT-cells or IL-17-producers γδT-cells in the CNS of EAE mice receiving WT versus DRD5KO DCs (Figure S4 in Supplementary Material). Moreover, we analysed whether DRD5-signalling in DCs was affecting the participation of T-cell subsets with suppressive activity in EAE, including Foxp3^+^ CD4^+^ Tregs, as well as LAP^+^, CD122^+^, and CD28^−^ CD8^+^ T-cells (21–23). We found no differences in the frequency of any of these suppressive T-cell subsets during the recovery phase and only a reduced frequency in Foxp3^+^ CD4^+^ Tregs infiltrating the CNS during the peak of the disease manifestation when mice lack DRD5-signalling in DCs (Figure S5 in Supplementary Material). Nevertheless, this latter difference was irrelevant as the extent of γδT-cells, which inhibit the Tregs function during the peak of the disease (18), was not affected by DRD5-signalling in DCs (Figure S4 in Supplementary Material). Thereby, the overall effect of DRD5-signalling in DCs was to favour the participation of inflammatory CD4^+^ T-cell subsets in the CNS of EAE mice, especially in the recovery phase.

### The Pro-Inflammatory Effect of DRD5-Signalling in DCs Is Mediated by the Inhibition of STAT3 Phosphorylation

**Figure 2:**
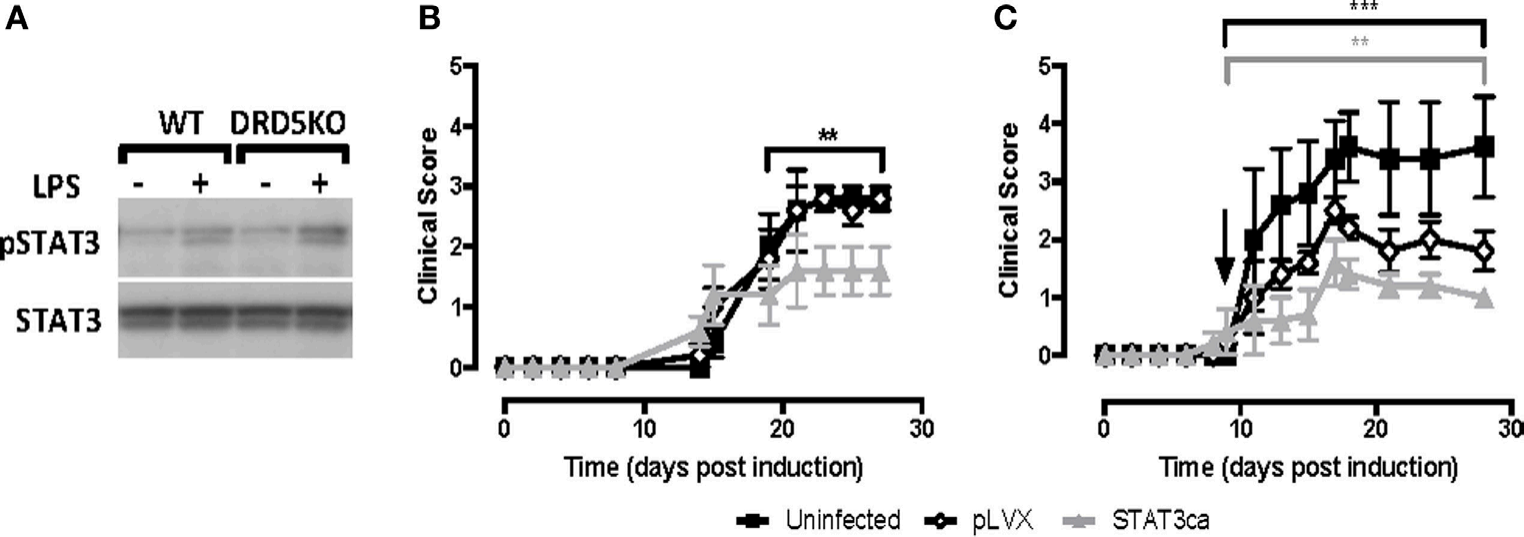
DRD5-signalling in dendritic cells (DCs) involves attenuation of signal transducer and activator of transcription 3 (STAT3) phosphorylation and the transduction of DCs with a constitutively active version of STAT3 exerts a therapeutic effect in experimental autoimmune encephalomyelitis (EAE). **(A)** Wild type (WT) or DRD5-knockout (DRD5KO) bone marrow-derived DCs (BM-DCs) were left unstimulated or treated with lipopolysaccharide (LPS) (250 ng/ml) for 8 h. Cells were lysed in the presence of phosphatase inhibitors, and the presence of phosphorylated STAT3 (pSTAT3, top panel) or total STAT3 irrespective of phosphorylations (STAT3, bottom panel) were analysed in protein extracts by Western blot. Representative data from one out of three independent experiments is shown. Not edited blots are shown in Figure S13 in Supplementary Material. **(B,C)** WT BM-DCs were left uninfected (black symbols), transduced with an empty lentiviral vector (pLVX, open symbols) or transduced with a lentiviral vector expressing a constitutively active form of STAT3 (STAT3ca, grey symbols) and pulsed with pMOG. DCs were transferred (10^6^ DCs/mice; i.v. injections) into WT recipient mice at days 14 and 7 prior to EAE induction **(B)** or 10 days post-induction **(C)**. The time of the therapeutic treatment in panel **(C)** is indicated by an arrow. Disease severity was evaluated as clinical score (see Materials and Methods) from day 0 to day 25 post-induction. Data from six to eight mice per group, corresponding to a representative from three independent experiments, are shown. Values represent mean ± SEM. **p < 0.01; ***p < 0.001; by Mann–Whitney *U*-test in the indicated time frames. Significant differences between uninfected and treated with pLVX are indicated in black brackets while differences between infected with pLVX and infected with STAT3ca are indicated in grey brackets.

**Figure 3:**
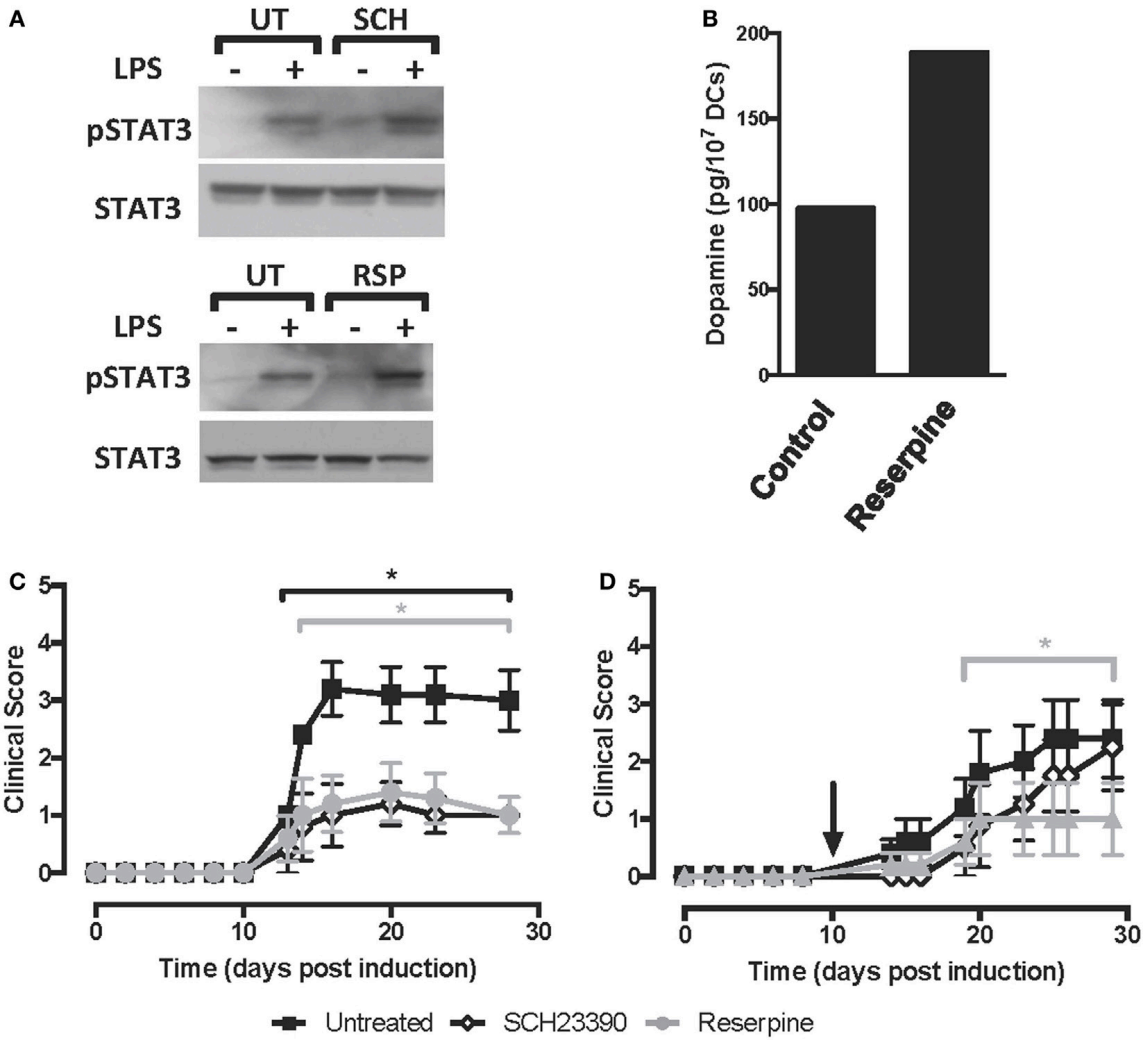
Pharmacologic inhibition of D5R-signalling or the depletion of dopamine on dendritic cells (DCs) reduces the severity of experimental autoimmune encephalomyelitis (EAE). **(A)** Wild type (WT) bone marrow-derived DCs (BM-DCs) were left untreated (UT) or treated with either the DRD1/DRD5 antagonist SCH23390 (SCH) or reserpine (RSP), alone or in the presence of lipopolysaccharide (LPS) (250 ng/ml) for 8 h. Cells were lysed in the presence of phosphatase inhibitors, and the presence of phosphorylated STAT3 (pSTAT3, top panel) or total signal transducer and activator of transcription 3 (STAT3) irrespective of phosphorylations (STAT3, bottom panel) was analysed in protein extracts by Western blot. Representative data from one out of three independent experiments are shown. Not edited blots are shown in Figure S13 in Supplementary Material. **(B)** WT DCs were either left unstimulated (control) or stimulated with 1 µM RSP for 60 min and then dopamine release was determined in the supernatant by an ELISA-based kit. A representative result from two independent experiments is shown. **(C,D)** WT BM-DCs were left untreated (black symbols), treated with SCH23390 1 µM (open symbols) or treated with RSP 1 µM (grey symbols) as described and pulsed with pMOG. DCs were transferred (10^6^ DCs/mice; i.v. injections) into WT recipient mice at days 14 and 7 prior to EAE induction **(C)** or 10 days post-induction **(D)**. The time of the therapeutic treatment in **(D)** is indicated by an arrow. Disease severity was evaluated as clinical score (see Materials and Methods) from day 0 to day 25 post-induction. Data from six to eight mice per group, corresponding to a representative from three independent experiments, are shown. Values represent mean ± SEM. *p < 0.05 by Mann–Whitney *U*-test in the indicated time frames. Significant differences between untreated and treated with SCH23390 are indicated in black brackets while differences between untreated and treated with reserpine (RSP) are indicated in grey brackets.

We previously described that DRD5-signalling in DCs favours selectively the production of both IL-23 and IL-12 (37). Since STAT3 has been involved in the repression of both of these cytokines in DCs (10, 11), we next evaluated whether DRD5-signalling in DCs involves the regulation of STAT3 activation. For this purpose, we induced STAT3 activation in DCs by stimulation with LPS (10) and then we analysed how DRD5-deficiency affected the extent of STAT3-phosphorylation by Western blot. These results show that lack of DRD5-signalling in DCs results in an exacerbated phosphorylation of STAT3 induced by LPS (Figure 2A), indicating that DRD5-signalling attenuates STAT3 activation in these cells. To determine the relevance of STAT3 activation in DCs in the development of EAE, we next transduced WT DCs with lentiviral vectors codifying for an STAT3ca (40) and GFP as reporter gene or with control vectors codifying only for GFP. Transduced cells were purified by cell sorting based on GFP-expression and then loaded *ex vivo* with pMOG and transferred into WT recipients in which EAE was induced. When STAT3ca-transduced DCs were transferred in a prophylactic regime to EAE mice, there was a significant attenuation of EAE manifestation in comparison with those animals receiving uninfected DCs or DCs transduced with control vectors (Figure 2B). Furthermore, when STAT3ca-transduced DCs were administered to EAE mice in a therapeutic context (day 10 post-induction), the disease severity was also strongly reduced (Figure 2C). To confirm the relevance of DRD5–STAT3 axis in DCs in favouring the development of EAE using a pharmacologic approach, we next performed a set of experiments using the D_1_-like-selective antagonist SCH23390. According to the genetic evidence indicating the role of DRD5-signalling in the inhibition of STAT3 activation (Figure 2A), when LPS-stimulated DCs were pre-incubated with SCH23390, an exacerbated STAT3-phosphorylation was observed (Figure 3A, top panel). Furthermore, when pMOG-loaded DCs were *ex vivo* treated with SCH23390 and then transferred into EAE mice in a prophylactic regime, we observed a decrease in disease severity (Figure 3C) in a similar extent of that reduction observed when DRD5KO DCs (Figure 1A) or STAT3ca-transduced DCs (Figure 2B) were transferred into EAE mice. Nevertheless, when pMOG-loaded SCH23390-treated DCs were transferred into EAE mice in a therapeutic context, there was no effect in disease severity (Figure 3D). Taken together, these results indicate that DRD5–STAT3 axis in DCs promotes the inflammatory response involved in EAE development.

### The Dopamine Produced by DCs Constitutes a Relevant Source to Trigger DRD5-Signalling

Previous studies have shown that DCs from mouse and human origin may produce and store dopamine in intracellular vesicles (36, 37). Moreover, *in vitro* analyses have suggested that toll-like receptor-stimulation and antigen-presentation are stimuli to induce dopamine release from DCs (36, 37). Thus, we next addressed the question of whether the dopamine produced by DCs is a relevant source for the autocrine stimulation of DRD5-signalling *in vivo* in the context of EAE. Accordingly, we performed a set of experiments using RSP, a drug that reverts the flow of the vesicular monoamine transporter, promoting the depletion of dopamine from secretory vesicles and redirecting it to the secretion to the extracellular compartment or degradation in the cytoplasm by the action of oxidative enzymes (29). Notably, when dopamine was depleted from DCs prior to LPS stimulation (Figure 3B), an exacerbated STAT3-phosphorylation was observed (Figure 3A, bottom panel), suggesting that dopamine contained inside DCs was required to DRD5-mediated inhibition of LPS-induced STAT3 activation. As expected, we observed that the treatment of DCs with RSP induced dopamine release into the culture supernatant (Figure 3B). To confirm the relevance of DCs as a source of dopamine to self-stimulate DRD5-signalling *in vivo*, we treated WT DCs with RSP *ex vivo*, which were then loaded with pMOG and transferred into EAE mice. Importantly, the results show that dopamine-depletion in DCs results in a significant reduction of EAE manifestation when pMOG-loaded DCs were administered in both contexts prophylactic or therapeutic (Figures 3C,D). Therefore, these results indicate that dopamine produced by DCs constitutes a relevant source of this mediator, which exerts an autocrine loop for the stimulation of DRD5-signalling *in vivo*.

### Dopamine Attenuates STAT3 Phosphorylation in Human Monocytes

**Figure 4:**
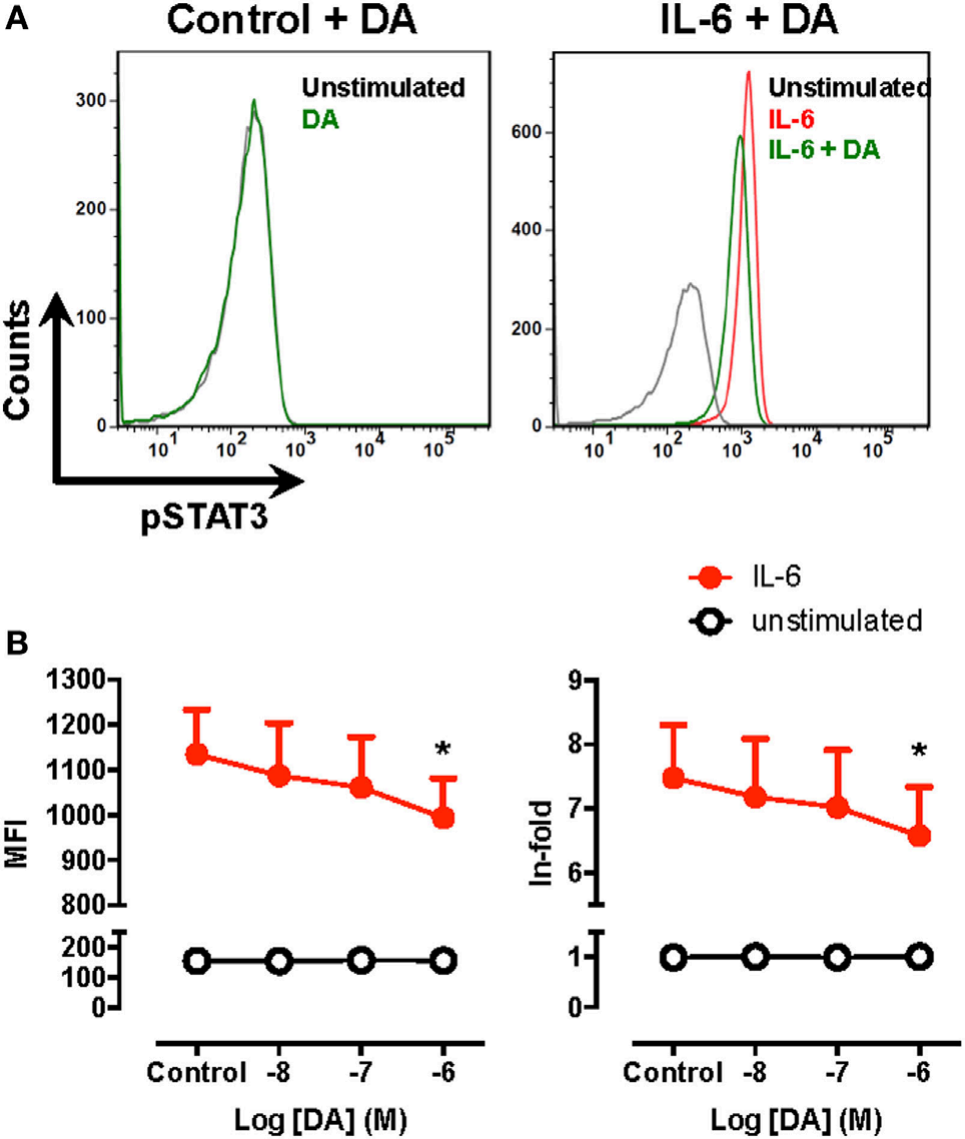
Signal transducer and activator of transcription 3 (STAT3) phosphorylation is decreased by dopamine in human monocytes. Fresh blood samples obtained from healthy donors were unstimulated or pre-incubated with dopamine (DA) in the presence or absence of interleukin (IL)-6 for 15 min. Afterwards, the extent of phosphorylated STAT3 (pSTAT3) was evaluated by intracellular immunostaining in the CD14^+^ population and analysed by flow cytometry using the gating strategy indicated in Figure S7 in Supplementary Material. **(A)** Representative histograms for pSTAT3 in peripheral blood CD14^+^ cells unstimulated (black lines) or treated with 1 µM DA (green lines) in the absence (left panel) or in the presence of IL-6 (right panel). The extent of pSTAT3 was also determined in peripheral blood CD14^+^ cells stimulated with IL-6 alone (red line). **(B)** The extent of pSTAT3 in unstimulated (white symbols) or IL-6 treated (black symbols) CD14^+^ in the presence of increasing DA concentrations was quantified as the mean fluorescence intensity (MFI) associated with pSTAT3-immunostaining (left panel) or as the ratio of the pSTAT3-associated MFI of stimulated cells to the pSTAT3-associated MFI of unstimulated cells (in-fold, right panel). Values represent mean ± SEM, *n* = 5. *p < 0.05 by unpaired two-tailed Student’s *t*-test.

In the above experiments, we used BM-DCs as a source of APCs, which have been described to be constituted by a heterogeneous mixture of myeloid APCs, including DCs, monocytes/macrophages, and cells expressing phenotypic markers of both DCs and monocytes/macrophages (47). In human, whereas monocytes constitute about 7–9% of PBMCs, DCs represent less than 1% (see Table 2). Since blood samples obtained were of a very limited volume, monocytes are significantly more abundant than DCs in human blood and they constitute a substantial fraction of APCs present in mouse BM-DCs (see CD14^+^ population in Figure S6 in Supplementary Material), we next evaluated whether STAT3 activation was also inhibited by dopaminergic stimulation in human monocytes. Accordingly, blood cells obtained from healthy controls were treated with IL-6, a stimulus previously described to induce STAT3 activation (48), and the extent of STAT3 phosphorylation was evaluated by flow cytometry in the CD14^+^ population using the gating strategy indicated (Figure S7 in Supplementary Material). Importantly, when monocytes were pre-incubated with increasing concentrations of dopamine, only 1 µM dopamine but not lower concentrations of dopamine, was able to reduce significantly the extent of STAT3-phosphorylation induced by IL-6 (Figure 4). Similar results were obtained when mouse BM-DCs were stimulated with IL-6 in the presence of 1 µM dopamine (Figure S8 in Supplementary Material). Thus, considering the affinities described for the binding of dopamine to the different DRs (27), these results suggest that the selective stimulation of DRD5-signalling in human monocytes is also coupled to the inhibition of STAT3 activation.

**Table 2:** Complete blood count.

	Units	Range	HS^a^	MS patients^a^	*p*
RBC	10^12^/L	4.5–6.0	4.6 ± 0.4	4.8 ± 0.4	0.400
Haemoglobin	g/dL	13.0–17.5	13.8 ± 1.2	14.3 ± 1.2	0.452
Platelets	10^9^/L	150–450	254.3 ± 61.7	218.4 ± 15.2	0.158
WBC	10^9^/L	4.3–11.0	6.0 ± 0.9	6.1 ± 2.0	0.860
Neutrophils	10^9^/L	1.5–5.5	3.5 ± 0.7	3.8 ± 1.7	0.674
	%	40–80	58.1 ± 5.1	60.4 ± 10.2	0.557
Lymphocytes	10^9^/L	1.5–5.5	1.9 ± 0.3	1.7 ± 0.8	0.420
	%	10–45	32.3 ± 5.1	28.0 ± 9.6	0.268
N/L			1.9 ± 0.4	2.5 ± 1.1	0.151
Monocytes	10^9^/L	0.2–1.1	0.4 ± 0.1	0.5 ± 0.2	0.194
	%	2–12	6.8 ± 1.6	8.9 ± 3.4	0.131

**Monocyte subsets**					
Classical	10^9^/L		0.31 ± 0.12	0.36 ± 0.14	0.495
	% of monocytes		73.8 ± 18.0	70.8 ± 7.5	0.651
Intermediate	10^9^/L		0.05 ± 0.01	0.09 ± 0.03	0.009
	% of monocytes		13.2 ± 2.5	16.2 ± 5.2	0.136
Non-classical	10^9^/L		0.03 ± 0.02	0.07 ± 0.04	0.023
	% of monocytes		8.2 ± 2.6	13.5 ± 5.8	0.022
DC	10^9^/L		0.017 ± 0.005	0.017 ± 0.007	0.864
	% of PBMC		0.8 ± 0.2	0.8 ± 0.2	1.000

**DC subsets**					
pDC	10^9^/L		0.005 ± 0.002	0.004 ± 0.001	0.262
	% of DC		27.0 ± 10.3	26.0 ± 8.7	0.825
mDC	10^9^/L		0.007 ± 0.001	0.008 ± 0.004	0.715
	% of DC		45.3 ± 11.4	44.3 ± 12.8	0.873

*Data are mean ± SD*.

*RBC, red blood cells; WBC, white blood cells; N/L, neutrophils-lymphocytes ratio; PBMC, peripheral blood mononuclear cells; HS, healthy subjects; MS, multiple sclerosis; mDC, myeloid DC; pDC, plasmacytoid DC*.

*^a^Data are missing for one HS and two MS patients*.

### MS Patients Display a Selective Overexpression of DRs in Peripheral Blood Inflammatory Monocytes

**Figure 5:**
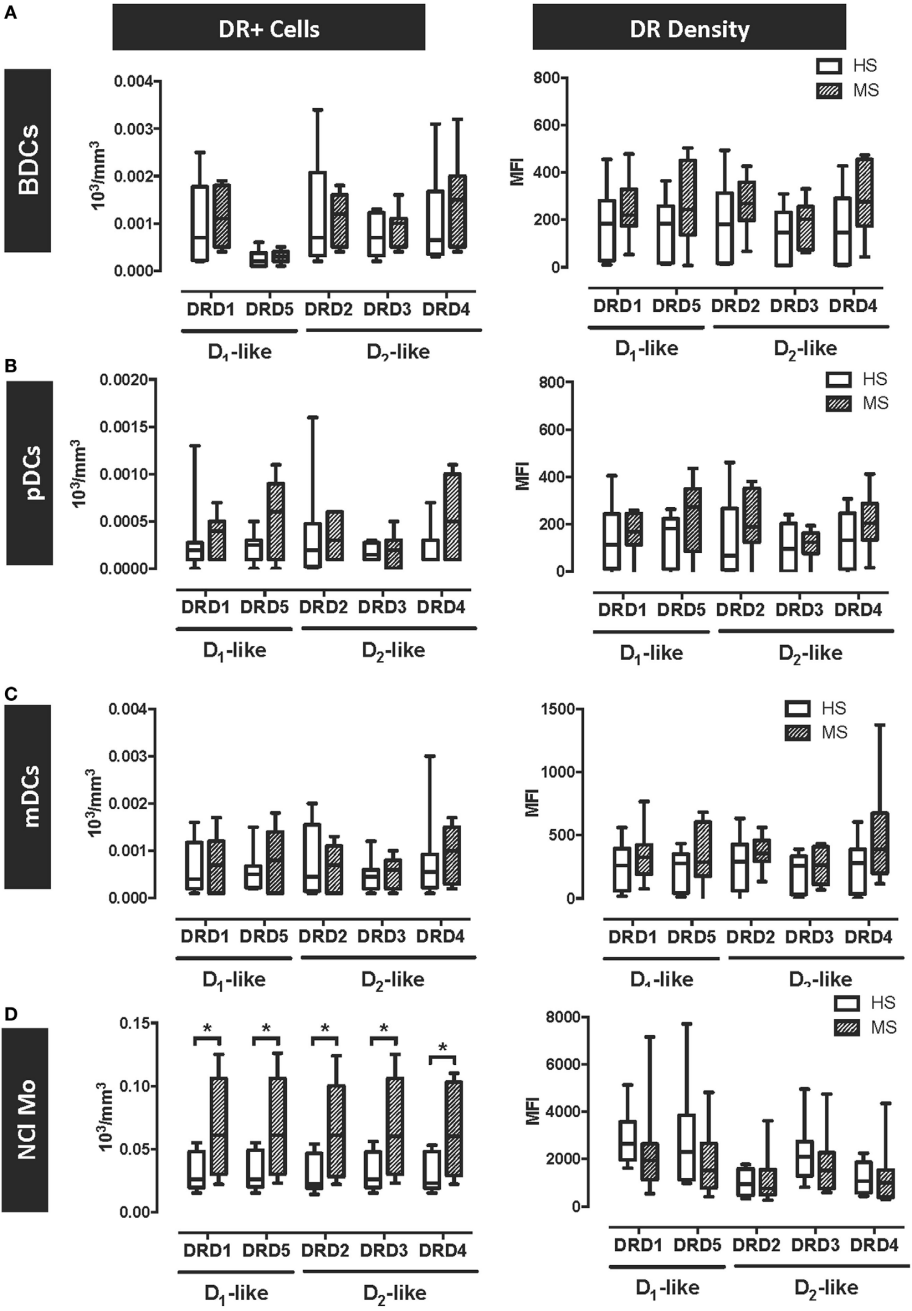
Dopamine receptor D5 (DRD5) expression is selectively increased in peripheral blood plasmacytoid and myeloid dendritic cells (DCs) obtained from MS patients. Fresh blood samples were obtained from healthy subjects (HS, *n* = 10; white bars) or multiple sclerosis patients (MS, *n* = 9; striped bars), and the expression of DRs (D_1_-like and D_2_-like) was analysed in different cell populations as the numbers of cells expressing each receptors (left panels) or the MFI associated with the immunostaining of each receptor (right panels). The expression of DRs in total peripheral blood DCs (BDCs) **(A)**, plasmacytoid DCs (pDCs) **(B)**, and myeloid DCs (mDCs) **(C)** were analysed based on the gating strategy described in Figure S9 in Supplementary Material. The expression of DRs in non-classical monocytes (NCl-Mo) **(D)** were analysed based on the gating strategy described in Figure S10 in Supplementary Material. Values represent mean ± SEM. *p < 0.05; **p < 0.01 by unpaired two-tailed Student’s *t*-test.

The results above indicate that DRD5–STAT3 axis in APCs promotes the inflammatory response associated with EAE in mouse and suggest that DRD5–STAT3 axis in APCs is also present in human. According to this evidence, we next wanted to evaluate whether DRD5-signalling in APCs was altered in MS patients in comparison to HS. For this purpose, we analysed the expression of all five DRs in several APCs populations present in human peripheral blood, including different subsets of DCs and monocytes. Whereas DCs subsets analysed included pDCs, mDCs, and total peripheral blood DCs (BDCs) (49), the analysis in monocytes was dissected in classical monocytes (Cl-Mo, described to exert phagocytic and non-inflammatory function), non-classical monocytes (NCl-Mo, associated to inflammatory and APC function), and intermediate monocytes (Int-Mo, a transitional subset displaying phagocytic and inflammatory features) (50). DRs expression was analysed in all these APCs populations by flow cytometry using the gating strategy described in the supplementary material for DCs (Figure S9 in Supplementary Material) and for monocytes (Figure S10 in Supplementary Material). The study enrolled 10 HS and 9 MS patients (Table 1). As shown in Table 2 and Figure S11 in Supplementary Material, the subsets of DCs analysed in HS and MS patients did not differ as both absolute numbers and percentage with respect to total PBMCs. Interestingly, the results show that DRs expression was not different between MS patients and HC in any of the subsets of DCs analysed, both at the level of number of cells expressing the receptors or at the level of DRs density in the cell surface (Figures 5A–C). Nevertheless, the results show an important increase in the absolute number of NCl-Mo expressing all five DRs in the cell surface (Figure 5D). This increase in NCl-Mo expressing DRs was associated with enhanced counts of NCl-Mo in peripheral blood and an augmented proportion of this monocyte subset in the total population of peripheral blood monocytes (Table 2). Moreover, the increased number of monocytes expressing DRs was selective to NCl-Mo, as the counts for Cl-Mo and Int-Mo expressing DRs did not differ between HC and MS patients (Figure S11 in Supplementary Material). The density of surface DRs expression was similar between HC and MS patients in all the monocytes subsets analysed (Figure 5D; Figure S12 in Supplementary Material). Thus, the overall analysis of DRs expression in APCs obtained from human individuals indicates a selective increase of the inflammatory subset of monocytes expressing all DRs in MS patients.

## Discussion

Early studies addressing the role of dopamine in the physiopathology of MS showed deregulated production of catecholamines and altered expression of a number of components of the dopaminergic system in PBMCs obtained from MS patients (33, 34). Subsequent studies analysing the role of dopaminergic alterations in MS patients confined to T-cells revealed an association of MS manifestation with the capability of Tregs to produce and respond to dopamine in a DRD5-dependent manner (35). In this regard, it has been described that human Tregs are capable to produce dopamine and, upon release of this mediator, suppressive activity is abolished by a DRD5-dependent mechanism (51). Importantly, both DRD5 expression and the capability to synthesise dopamine are exacerbated in untreated MS patients, thus reducing the anti-inflammatory function of Tregs. However, it has been shown that the treatment of MS patients with IFN-β reduces the levels of DRD5 and abolishes the expression of the machinery required to synthesise dopamine in Tregs, thus re-establishing their suppressive activity (35). Thereby, Tregs seem to be key players in the dopaminergic regulation of inflammation in the physiopathology of MS. In the present study, we described myeloid APCs as another key player of the dopaminergic regulation of immunity, which seems to play an important role promoting the inflammatory response in EAE and MS by a DRD5-dependent mechanism. Of note, our findings described here together with previous evidence indicate that DRD5-driven effects in different types of immune cells promote inflammation, supporting the idea that DRD5 results a key molecular target to limit autoimmunity.

It is important to consider that in our study, we included just a small sample size of MS patients and healthy individuals, which had the purpose of exploring whether the pro-inflammatory effect of DRD5-signalling observed in mouse APCs in the context of EAE was equivalent in human APCs obtained from MS patients. Due to the high difficulty of obtaining samples from untreated MS patients, we used here blood samples obtained from IFN-β-treated patients. To rule out the possibility that IFN-β treatment affected the frequency of different subsets of myeloid cells and/or the expression of functionally relevant molecules in these cells, we analysed these parameters and observed similar frequencies of DCs subpopulations and expression of HLA-DR when samples from MS patients were compared with HS (Figures S11 and S12 in Supplementary Material; Table 2). The only significant difference detected in our analyses carried out in blood samples obtained from MS patients was that their inflammatory subset of monocytes was increased and displayed a stronger frequency of DRs expression in comparison with healthy individuals (Figure 5D). It is noteworthy that this observation agrees with the pro-inflammatory role of DRD5-signalling described here in mouse myeloid APCs in the context of EAE.

In agreement with our findings described here, a previous study demonstrated that the systemic administration of the D_1_-like antagonist SCH23390 reduces the clinical manifestation of EAE in SJL/J mice immunised with a peptide derived from myelin proteolipid protein (52). Similarly, subsequent studies have shown that D_1_-like antagonism attenuates the inflammatory response in collagen-induced arthritis (53, 54), allergic asthma (55, 56), diabetes (57), and nephrotoxic serum nephritis (58). Mechanistic analyses have suggested that the therapeutic effect exerted by SCH23390 in these inflammatory disorders is due to D_1_-like antagonism in both CD4^+^ T-cells and DCs. The evidence suggests that D_1_-like DRs-signalling in CD4^+^ T-cells promotes B-cell activating transcription factor activity, favouring RAR-related orphan receptor gamma upregulation and consequently Th17-mediated responses, which is inhibited by SCH23390 (56). Conversely, the molecular mechanism by which this drug attenuates the inflammatory potential of DCs has been poorly explored. We demonstrated that the anti-inflammatory effect of D_1_-like antagonism in DCs was due to the inhibition of DRD5-signalling, which is coupled to the attenuation of STAT3 phosphorylation (Figure 3) and the consequent potentiation in IL-12 and IL-23 production (37).

Here, we show that DRD5-signalling dampens the phosphorylation of STAT3 triggered by LPS stimulation in DCs (Figures 2–4). Importantly, STAT3-activation has been involved in the repression of both IL-12 and IL-23 (10, 11). In agreement with these observations, our previous results have shown that DRD5-signalling in DCs favours selectively the production of the inflammatory cytokines IL-12 and IL-23, without effect in the secretion of IL-10, IL-6, IL-1β, and TGF-β (29, 37). Furthermore, in the present study our data indicate that DRD5-signalling confined to DCs strengthens Th1- and Th17-mediated immunity and the participation of GM-CSF-producing CD4^+^ T-cells *in vivo* (Figure 1), responses that depend on IL-12 and IL-23 activity (6, 7, 14). It is noteworthy that even if we trigger EAE development by immunisation with pMOG/CFA, the phenotype of encephalytogenic T-cells was evaluated by intracellular cytokine staining of T-cells after *ex vivo* restimulation by a polyclonal stimulus (PMA plus ionomycin) instead an antigen-specific stimulus (pMOG). In this regard, is has been shown that chronic progression of EAE is invariably linked to the process of epitope-spreading, which involves a shift of T-cell specificity from the initial immunogen (pMOG in this case) to others relevant CNS-derived auto-antigens (59). Thereby, to avoid an underestimation of the frequency associated with each encephalytogenic T-cell subset, we analysed polyclonal T-cells rather than pMOG-specific T-cells infiltrating the CNS.

Regarding the signalling pathways coupled to DRD5 in DCs, in a previous report we showed that DRD5-stimulation in DCs was coupled to the inhibition of ERK1/2-activation triggered by LPS (37). To address how DRD5-signalling was related with the decrease of ERK1/2-activation and the attenuation of STAT3-phosphorylation, we determined whether DRD5-stimulation was coupled to cAMP production in DCs, as cAMP has been involved in the attenuation of both ERK1/2 and STAT3 pathways in breast cancer cells (60, 61). In agreement with most reports addressing the coupling of DRD5 in different cell types (46), our results suggest that DRD5-stimulation in DCs was also coupled with the induction of cAMP production (Figure S1 in Supplementary Material). Thereby, it is tempting to speculate that cAMP triggered by DRD5-stimulation in DCs is a mediator responsible for the down-stream inhibition of ERK1/2 and STAT3 activation observed. In addition, previous studies have shown that ERK1/2 may potentiate the activation of STAT3 (62), suggesting that DRD5-mediated inhibition of ERK1/2 may also contribute in the reduction of STAT3-phosphorylation observed when DRD5 is stimulated in DCs.

Despite it is likely that LPS and IL-6 lack relevance as inducers of STAT3-activation in DCs in the *in vivo* context of EAE and MS, they are useful tools for *in vitro* experiments. Conversely, it is more probable that IL-10 produced by suppressive subsets of lymphocytes as well as CTLA4 interaction with B7 molecules are more relevant actors as inducers of STAT3-activation in the context of EAE and MS. In this regard, IL-10 produced by Tregs or by a suppressive subset of B-cells, B10 cells, has been shown to induce STAT3-activation in DCs and to play a relevant anti-inflammatory role dampening EAE manifestation (10, 63, 64). On the other hand, it has been reported that the B7-ligand CTLA4, which is expressed in Tregs surface, triggers STAT3-phosphorylation in DCs inducing a tolerogenic behaviour (65). In addition, it has been described that activated STAT3 may inhibit the recruitment of NF-κB to the promoter of p40 (10), a common subunit for IL-12 and IL-23. Moreover, it has also been reported that STAT3 activation may prevents the recruitment of the positive transcription elongation factor b to the promoter of IL-12p35 (11). Thus, previous evidence agrees with the tolerogenic effect of STAT3 observed here in DCs in the context of EAE.

An intriguing question about of dopaminergic-mediated regulation of immunity is what is the relevant source of this neurotransmitter to exert an effect on immune cells. In this regard, it is necessary to consider the spatiotemporal redistribution of immune cells during the development of the disease. In the case of EAE, APCs play important roles twice during the time-course of the disease, being involved first in the peripheral activation of CD4^+^ T-cells and later in the re-stimulation of T-cells inside the CNS (66). It is noteworthy that in the CNS, CD4^+^ T-cells may be restimulated by resident APCs, such as astrocytes or microglia, as well as by APCs infiltrating from the periphery such as DCs and monocytes/macrophages (67). Moreover, a previous work has shown a rise of dopamine levels in the striatum during the onset and the peak of EAE manifestation (32). Despite there is not direct evidence showing that DCs might infiltrate the striatum during the onset or the peak of EAE manifestation, this data suggests that dopamine produced in the CNS could represent a relevant source for the dopaminergic regulation of the immune response involved in this disorder. On the other hand, previous studies described that DCs can synthesise and store dopamine (29, 36, 37), thus suggesting that these cells may represent a source of this mediator available for immune cells in close proximity, including themselves. Here, we addressed the question of whether dopamine contained inside DCs is a relevant source of this neurotransmitter to trigger the pro-inflammatory behaviour of DCs mediated by DRD5-signalling. Our results here show a relevant and potent effect of DCs-derived dopamine in the stimulation of DRD5–STAT3 axis *in vivo*, favouring the development of EAE (Figure 3). Nevertheless, these results does not rule out the possibility that the rise of dopamine in the CNS during the onset and the peak of EAE manifestation plays a relevant role in the regulation of immunity during the development or progression of the disease. Furthermore, increasing evidence has demonstrated a constitutive activity of G-protein coupled receptors, including DRD5, which exerts physiologically relevant roles even in the absence of endogenous ligands (68). Thereby, these studies suggest that basal signalling triggered by DRD5 could contribute to the pro-inflammatory behaviour in DCs irrespective of the availability of dopamine for these cells.

In conclusion, the present study suggests a mechanism by which DRD5-signalling in myeloid APCs strengthens the inflammatory response mediated by self-reactive CD4^+^ T-cells in EAE. This mechanism involves an autocrine loop in which DCs-derived dopamine stimulates DRD5-signalling and the down-stream attenuation of STAT3-activation. Our data indicate that targeting this mechanism at different levels, a significant therapeutic effect was reached, including the depletion of DCs-derived dopamine, DRD5-antagonism or desensitising STAT3-inhibition in DCs. Furthermore, the analysis performed in human individuals indicated a selective and significant increase of dopaminergic signalling in inflammatory monocytes in MS patients. Thus, these results suggest an equivalent pro-inflammatory role of DRD5–STAT3 axis exerted by BM-DCs in EAE in mouse and by inflammatory monocytes in MS in human. Taken together, our findings describe a novel therapeutic target to dampen the inflammatory T-cell response in EAE with promising clinical potential for MS.

## Ethics Statement

The study performed with human individuals conforms to the principles outlined in the Declaration of Helsinki, the study protocol was approved by the local Ethics Committee of the Ospedale di Circolo and Fondazione Macchi, Varese (Italy). All procedures performed in animals were approved by and complied with regulations of the Institutional Animal Care and Use Committee at Fundación Ciencia & Vida.

## Author Contributions

MC and RP designed the study. CT, MG, HG, VT, AQ, and FO-B conducted experiments. CT, HG, VT, AQ, and ER acquired data. CT, MG, FM, MZ, MC, and RP analysed data. AL and EC provided new reagents. MC and RP wrote the manuscript.

## Conflict of Interest Statement

The authors declare that the research was conducted in the absence of any commercial or financial relationships that could be construed as a potential conflict of interest.

## Abbreviations

Ab, antibody; BM, bone marrow; CNS, central nervous system; CFA, complete Freund’s adjuvant; Cl-Mo, classical monocytes; DCs, dendritic cells; DR, dopamine receptor; DRD5, DR D5; DRD5KO, DRD5-knockout; EDSS, expanded disability status score; EAE, experimental autoimmune encephalomyelitis; GFP, green fluorescent protein; GM-CSF, granulocyte-macrophage colony-stimulating factor; HS, healthy subjects; HLA-DR, human leucocyte antigen DR; HRP, horseradish peroxidase; IFN-*n*, interferon *n*; IL-*n*, interleukin *n*; Int-Mo, intermediate monocytes; LAP, leucocyte alkaline phosphatase; LPS, lipopolysaccharide; mAb, monoclonal antibody; MFI, mean fluorescence intensity; MOG, myelin oligodendrocyte glycoprotein; mDCs, myeloid DCs; NCl-Mo, non-classical monocytes; NF-κB, nuclear factor kappa B; PRRs, pattern-recognition receptors; pMOG, peptide MOG; PBMC, peripheral blood mononuclear cells; BDCs, peripheral blood DCs; pDCs, plasmacytoid DCs; PE, phycoerythrin; PMA, phorbol 12-myristate 13-acetate; pSTAT3, phosphorylated STAT3; RORγt, RAR-related orphan receptor gamma; RSP, reserpine; SCH, SCH23390; STAT3, signal transducer and activator of transcription 3; STAT3ca, constitutively active STAT3; Tregs, regulatory T-cells; Th*n*, T helper *n*; TLRs, toll-like receptors; WT, wild type.
