# Supplementary material for: Dopaminergic Stimulation of Myeloid Antigen-Presenting Cells Attenuates Signal Transducer and Activator of Transcription 3-Activation Favouring the Development of Experimental Autoimmune Encephalomyelitis
Source: Front Immunol. 2018 Mar 21;9:571. doi: 10.3389/fimmu.2018.00571 (PMC5871671; doi:10.3389/fimmu.2018.00571)
Supplement: Supplementary file 1 [file presentation_1.PDF]

Dopaminergic stimulation of myeloid antigen-presenting cells attenuates STAT3 activation favouring the development of experimental autoimmune encephalomyelitis

Carolina Prado, Michela Gaiazzi, Hugo González, Valentina Ugalde, Alicia Figueroa, Francisco Osorio-Barrios, Ernesto López, Alvaro Lladser, Emanuela Rasini, Franca Marino, Mauro Zaffaroni, Marco Cosentino, Rodrigo Pacheco

SUPPLEMENTARY MATERIAL

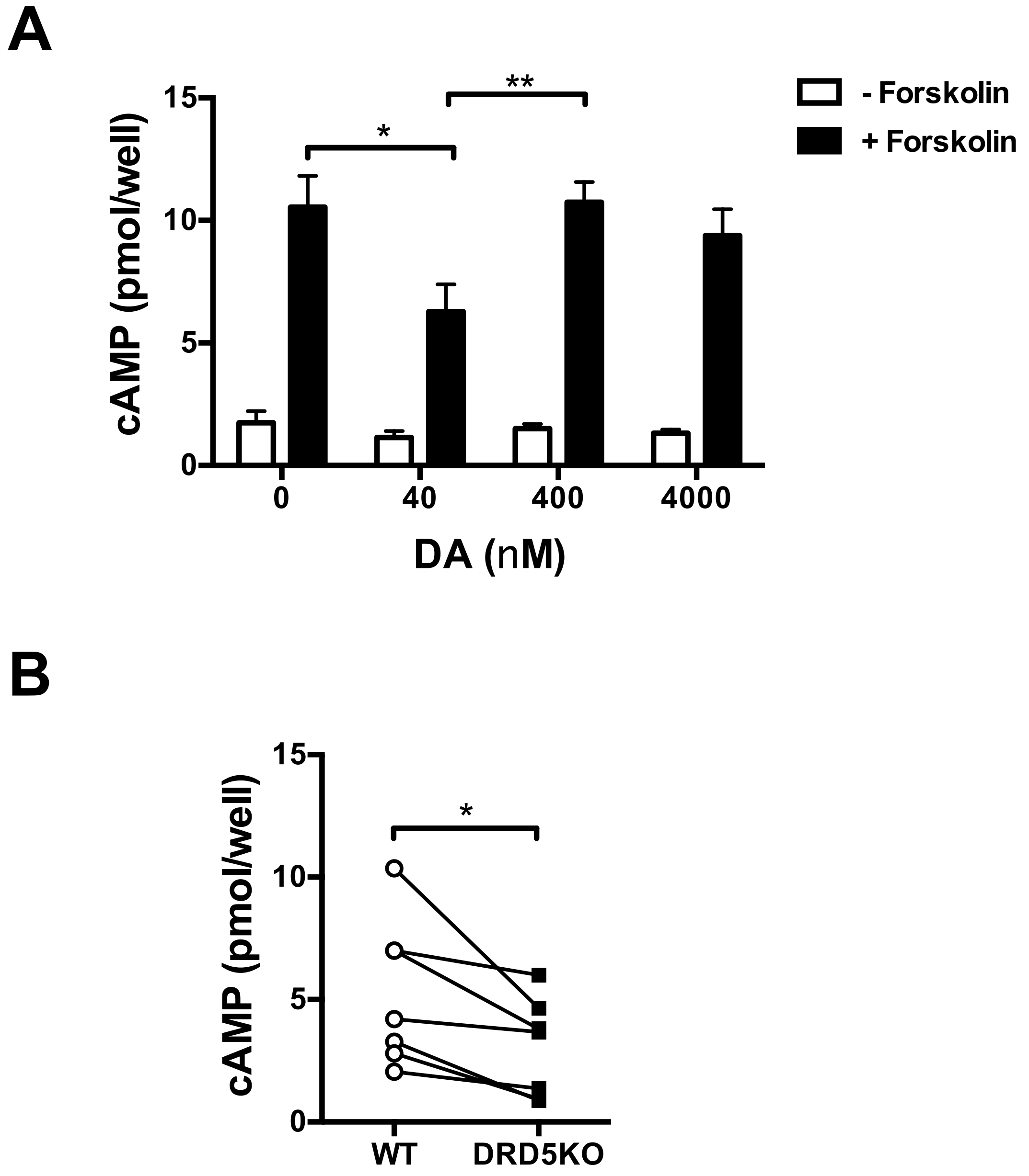

Figure S1. DRD5-signalling in DCs involves stimulation of cAMP production. (**A**) WT DCs (10^6/mL) were preincubated with the phosphodiesterase inhibitor zardaverine (50 nM) for 30min and then treated with dopamine (DA) at indicated concentrations in the presence (black symbols) or absence (white symbols) of the adenylyl cyclase activator Forskolin (500 nM) for additional 30min. Later, intracelullar levels of cAMP were determined by ELISA. Data represent mean ± SEM from 3 independent experiments. *, p<0.05; **, p<0.01 by unpaired Student's *t*-test. (**B**) WT (white symbols) or DRD5KO (black symbols) DCs were preincubated with zardaverine for 30min and then treated with forskolin for additional 30min. Afterward, intracellular levels of cAMP were determined by ELISA. Data represent mean ± SEM from seven independent experiments. *, p<0.05 by paired Student's *t*-test.

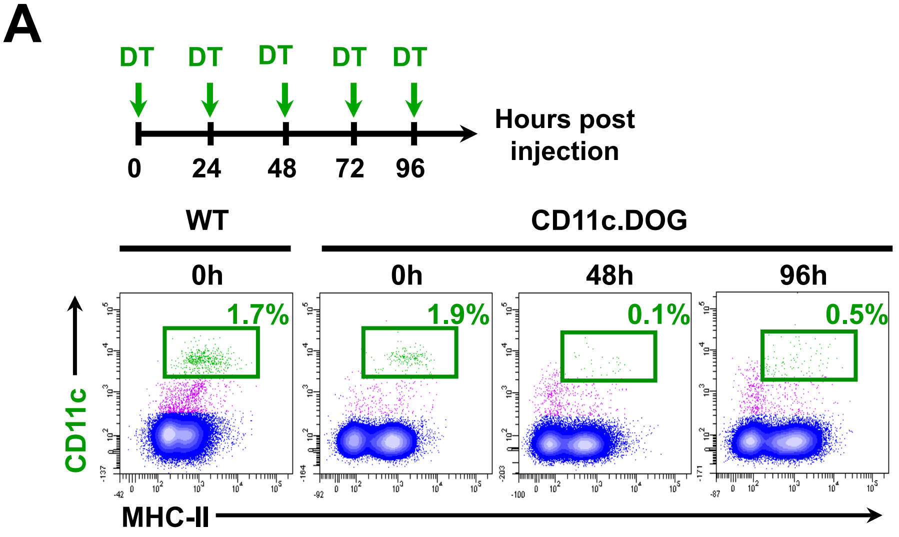

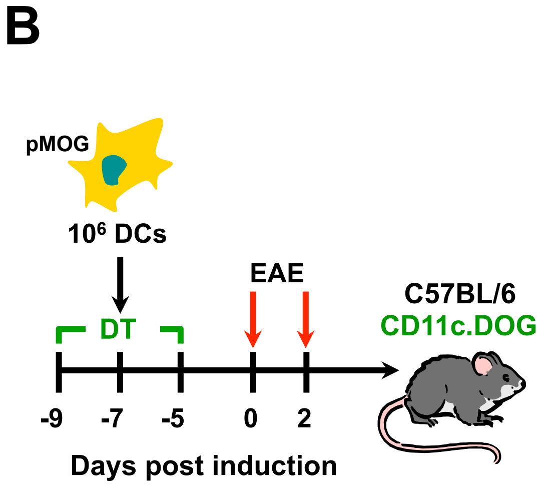

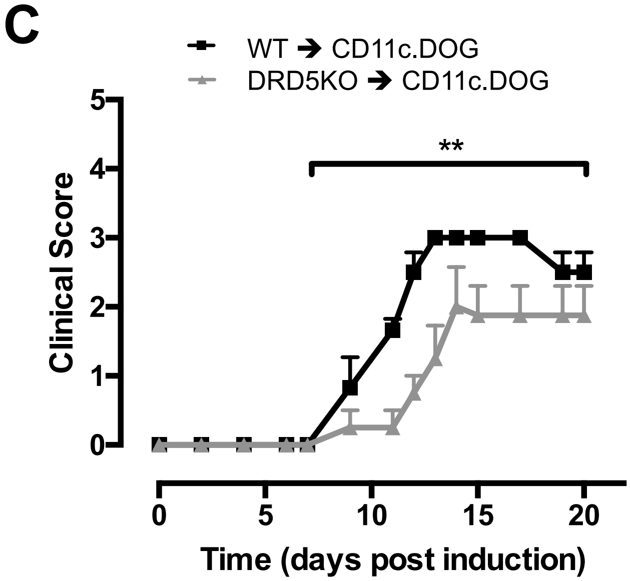

Figure S2. DRD5-deficient DCs loaded ex vivo with the autoantigen attenuates EAE development in recipient mice depleted of endogenous DCs. (**A**) CD11c.DOG mice received daily i.p. injections of diphteria toxin (DT; 8 ng per gram of body weight) and frequency of CD11c$^+$ MHC-II$^+$ DCs was evaluated in spleen at indicated times. The percentage of CD11c$^+$ MHC-II$^+$ cells was determined in the spleen of DT-treated CD11c.DOG and WT mice. Top panel illustrates a scheme of the time course of the treatment and bottom panel shows representative dot-plots indicating the frequency of splenic DCs (green region). (B and C) CD11c.DOG recipient mice received daily injections of DT to deplete endogenous DCs in the time frame from day 9 to day 5 prior EAE induction. WT (black line) or DRD5KO (grey line) DCs were ex vivo pulsed with pMOG and transferred (10^6 DCs/mice; i.v. injections) into DT-treated CD11c.DOG recipient mice at day 7 prior to EAE induction. (**B**) Scheme of the time course of the treatment. (**C**) Disease severity was evaluated as the clinical score from day 0 to day 20 post-induction. Data from at least 14 mice per group are shown, corresponding to a representative from three independent experiments. Values represent mean ± SEM. **, p<0.01 by Mann-Whitney U-test in the indicated time frames.

A

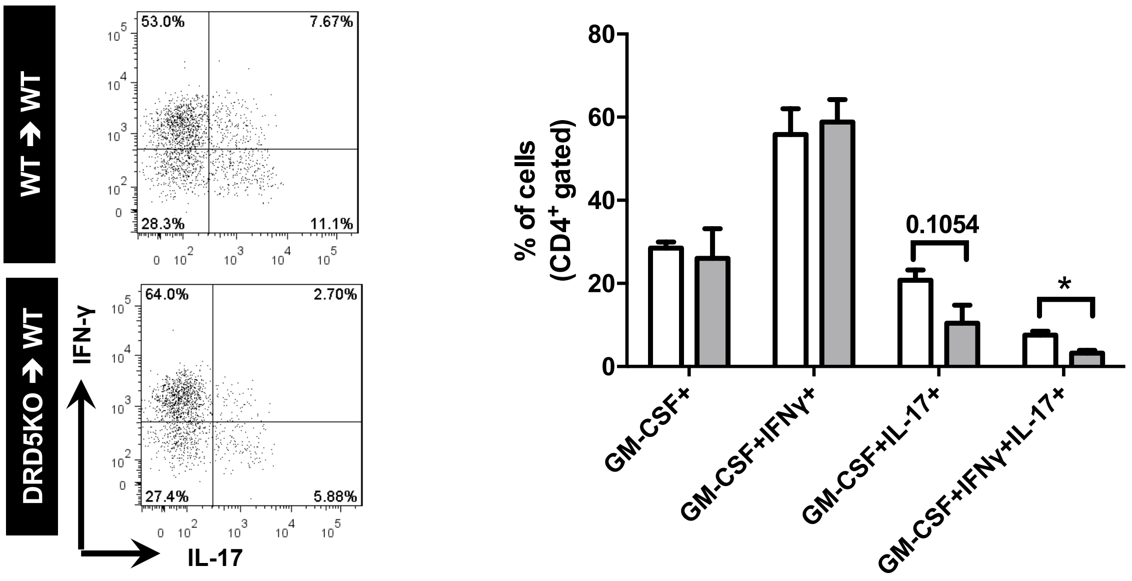

B

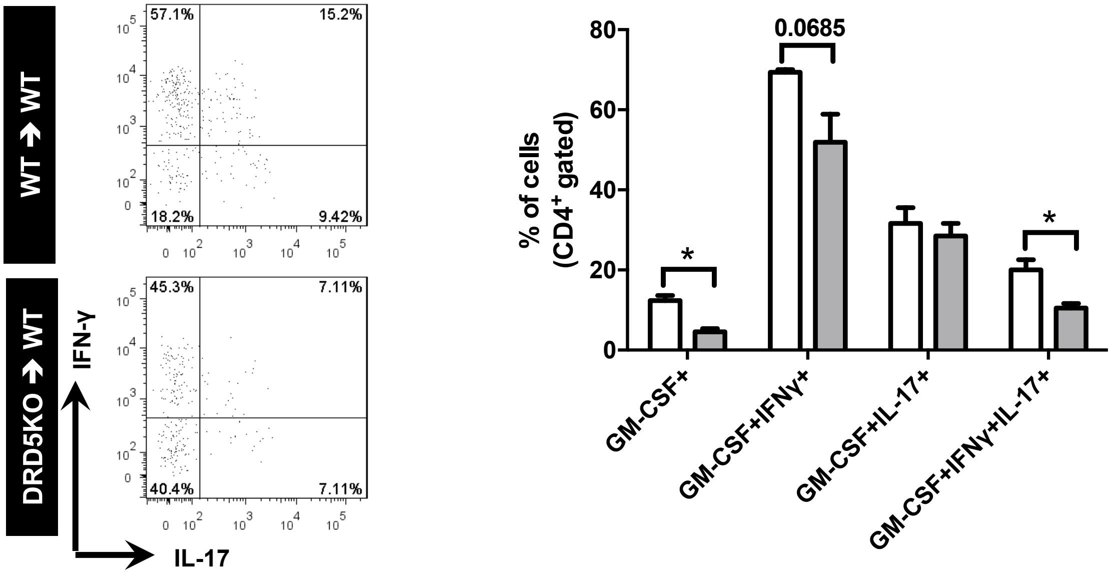

Figure S3. Deficiency of DRD5-signalling in DCs results in a decreased frequency of inflammatory CD4$^+$ T-cell subsets infiltrating the central nervous system of EAE mice. EAE was induced as indicated in figure 1A and, at the peak (17-19 dpi) or at the recovery phase (25 dpi) of the disease, mononuclear cells were isolated from CNS followed by *ex vivo* stimulation with PMA/ ionomycin in the presence of brefeldin A, and intracellular cytokine staining analysis was carried out by flow cytometry. Inflammatory T-cell subsets were analysed at the peak (**A**) or at the recovery phase (**B**). (A and B) Representative dot-plots for IL-17 and IFN-γ expression in the infiltrating CD4$^+$ GM-CSF+ gated population are shown in left panels. Numbers on the dot-plots indicate the percentage of cells in the associated region. Quantification of frequencies of inflammatory T-cells infiltrating the CNS are represented (right panels) as the mean ± SEM. *, $p<0.05$ by unpaired two-tailed Student's *t*-test.

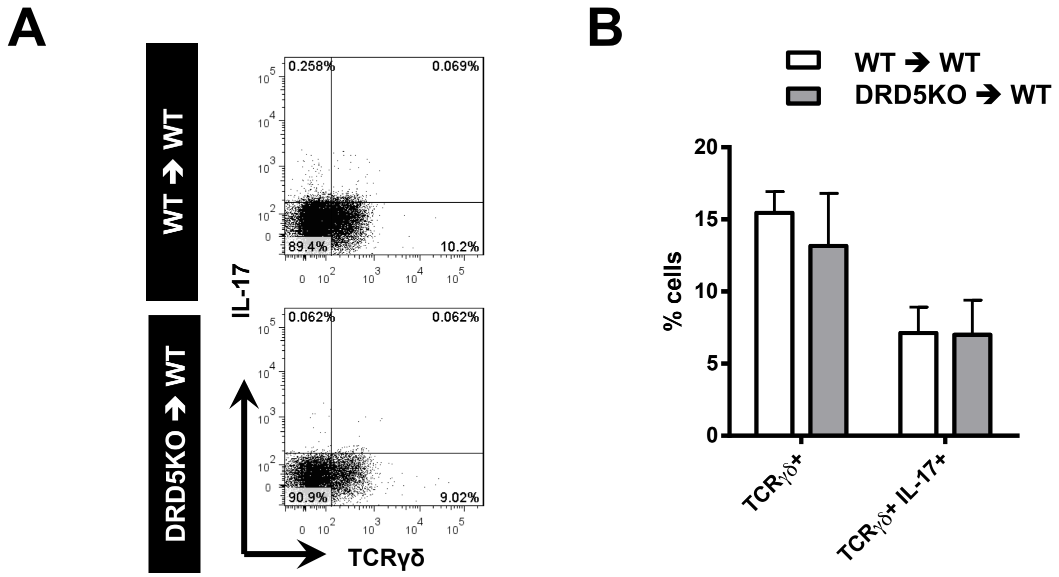

Figure S4. Deficiency of DRD5-signalling in DCs does not affect the frequency of TCRγδ infiltrating the central nervous system of EAE mice during the peak of disease manifestation. EAE was induced as indicated in figure 1A and, at the peak of the disease manifestation (17-19 dpi), mononuclear cells were isolated from CNS followed by *ex vivo* stimulation with PMA/ionomycin in the presence of brefeldin A, and intracellular cytokine staining analysis was carried out by flow cytometry. **(A)** Representative dot-plots for TCRγδ and IL-17 expression in the infiltrating $CD3^+$ $CD4^-$ $CD8^-$ gated population are shown. **(B)** Quantification of frequencies of TCRγδ T-cells (respect to the $CD3^+$ $CD4^-$ $CD8^-$ gate) and of IL-17-producers TCRγδ T-cells (respect to the $CD3^+$ $CD4^-$ $CD8^-$ $TCR\gamma\delta^+$ gate) infiltrating the CNS are represented as the mean ± SEM. Not significant differences were found.

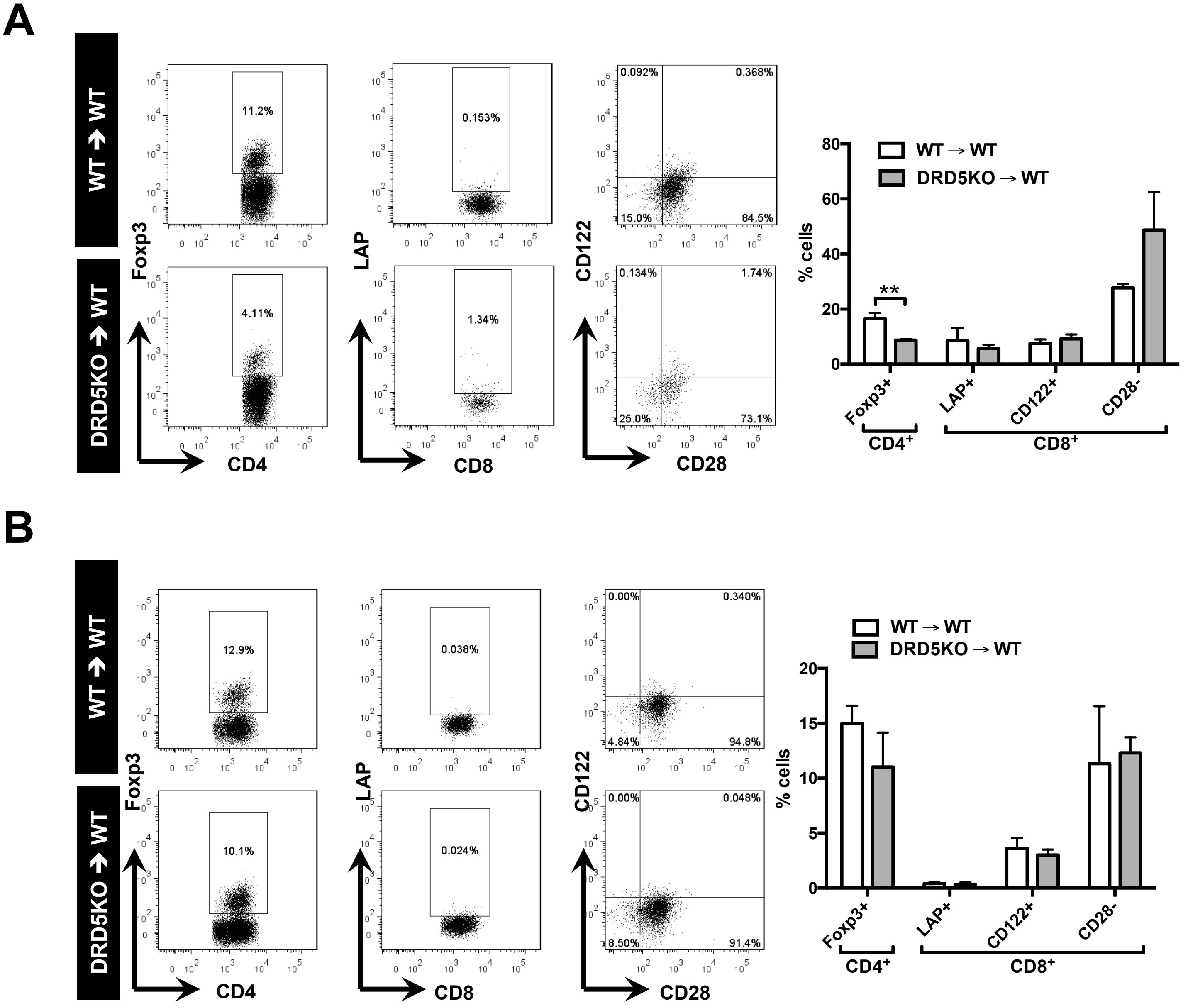

Figure S5. Deficiency of DRD5-signalling in DCs results in a decreased frequency of Foxp3$^+$ CD4$^+$ T-cells infiltrating the central nervous system of EAE mice only during the peak of disease manifestation but not in the recovery phase. EAE was induced as indicated in figure 1A and, at the peak (17-19 dpi) or at the recovery phase (25 dpi) of the disease, mononuclear cells were isolated from CNS followed by *ex vivo* stimulation with PMA/ionomycin in the presence of brefeldin A, and intracellular cytokine staining analysis was carried out by flow cytometry. Anti-inflammatory populations were analysed at the peak (**A**) or at the recovery phase (**B**). (A and B) Representative dot-plots for Foxp3 expression in the infiltrating CD4$^+$ gated population and LAP, CD28 and CD122 expression in the infiltrating CD8$^+$ gated population are shown in left panels. Numbers on the dot-plots indicate the percentage of cells in the associated region. Quantification of frequencies of anti-inflammatory T-cells infiltrating the CNS are represented (right panels) as the mean ± SEM. **, p<0.01 by unpaired two-tailed Student's *t*-test.

Figure S6., Prado et al.

A

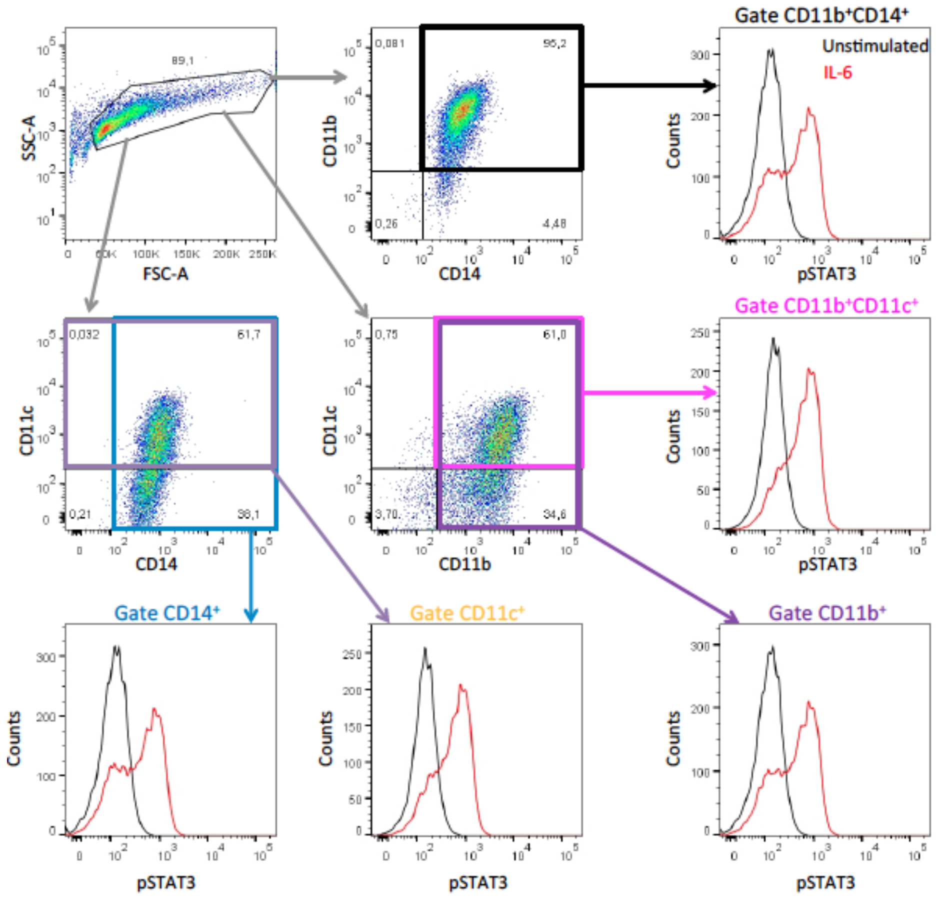

B

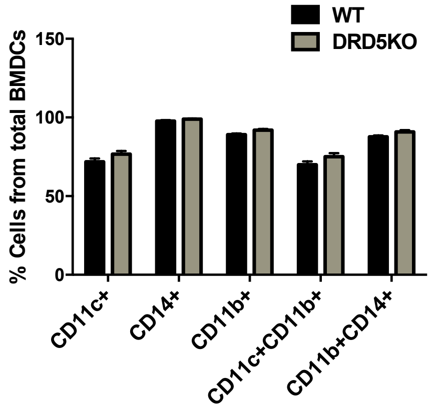

Figure S6: DCs obtained from bone marrow precursors of mice constitute an heterogeneous mix of DCs and macrophages. Bone marrow precursors obtained from WT and DRD5KO mice were differentiated to BMDCs with 10 ng/mL of GM-CSF for 6 days. Afterwards, cells were collected and the expression of CD11c, CD11b and CD14 was analysed by flow cytometry. (**A**) Gating strategy is shown with representative dot plots obtained for WT BMDCs. Numbers on the dot plots indicate the percentage of cells in the corresponding region. (**B**) Quantification of frequencies of BMDCs in each gate analysed. Data from seven independent experiments is shown. Values corresponds to mean with SEM. No significant differences were found between both genotypes analysed.

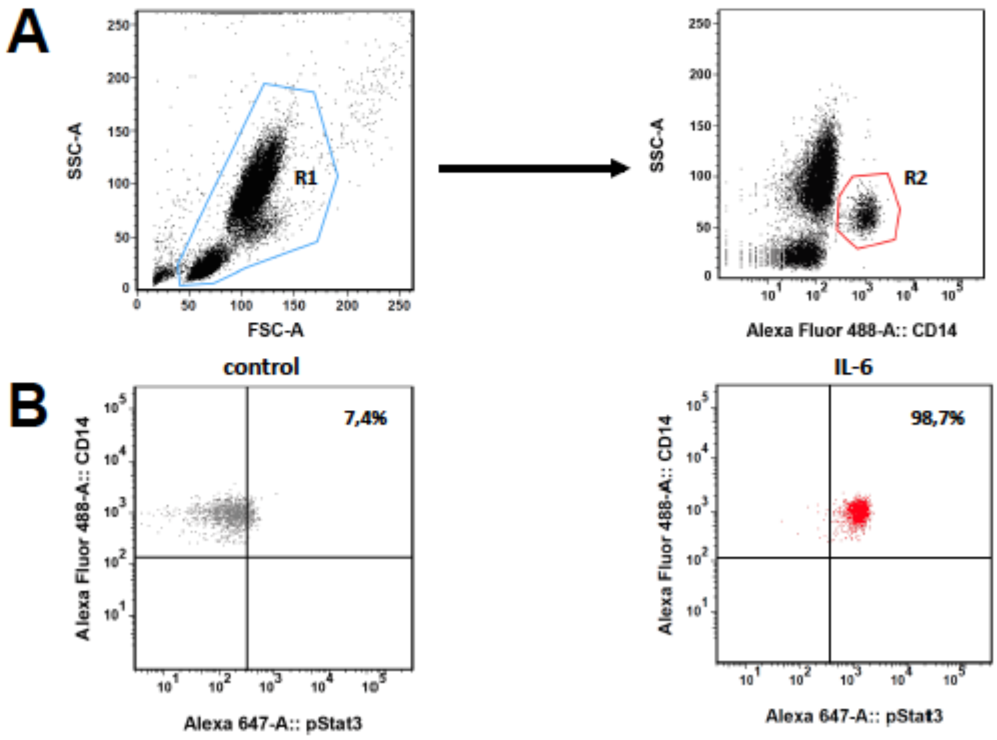

Figure S7. Gating strategy for the analysis of STAT3 posphorylation in human peripheral blood monocytes. Fresh blood samples obtained from healthy donors were left untreated (control) or stimulated with IL-6 for 15 min. Afterwards, the extent of phosphorylated STAT3 (pSTAT3) was evaluated by intracellular immunostaining in the CD14$^+$ population and analysed by flow cytometry. (**A**) Gating strategy to analyse the CD14$^+$ population. (**B**) representative dot-plots showing the immunostaining of pSTAT3 and CD14 in cells unstimulated (left panel) or treated with IL-6 as a positive control (right panel).

A

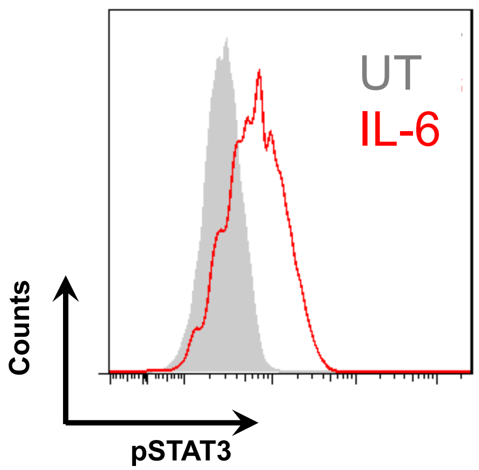

B

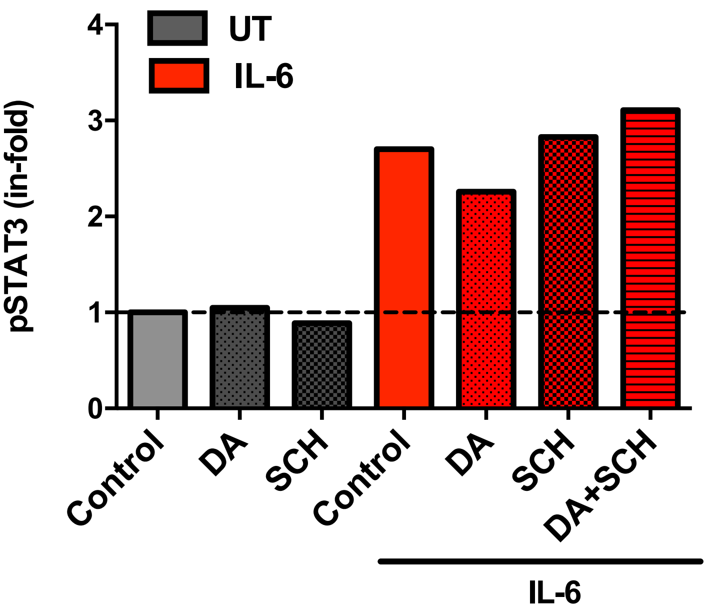

Figure S8. Dopamine attenuates STAT3 phosphorylation in mouse DCs. WT DCs were pre-treated for 5 minutes with 1μM SCH23390, 1μM DA or both together (SCH23390 added 5 mins before DA), and then left untreated (UT, grey) or stimulated with 10 ng/mL IL-6 (red). Afterwards, the extent of phosphorylated STAT3 (pSTAT3) was evaluated by intracellular immunostaining and analysed in the CD11c^{+} population by flow cytometry. **(A)** Representative histograms are shown. **(B)** Quantification of pSTAT3. Values correspond to the ratio of the pSTAT3-associated MFI of stimulated cells to the pSTAT3-associated MFI of unstimulated cells (in-fold).

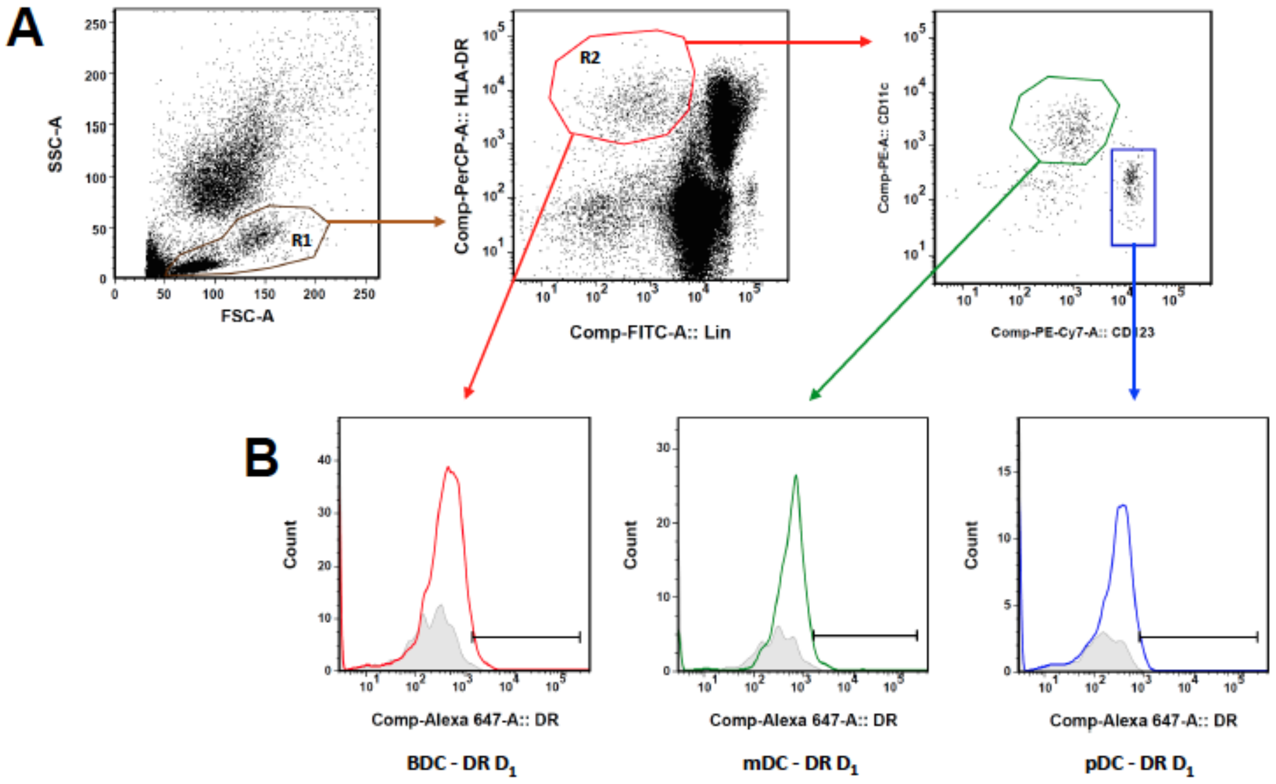

Figure S9. Gating strategy for the analysis of dopamine receptors in peripheral blood plasmacytoid and myeloid dendritic cells from human individuals. Fresh blood samples obtained from healthy donors were immunostained with antibodies specific to different DRs and to surface markers associated to myeloid ($CD11c^{high}$ $CD123^{low}$) plasmacytoid ($CD11c^{low}$ $CD123^{high}$) and total ($HLA\text{-}DR^{+}$ Lin^{-}) DCs and analysed by flow cytometry. (**A**) Representative dot-plots showing the gating startegy used for selection of total blood DCs (BDCs, red), myeloid DCs (mDCs, green) and plasmacytoid DCs (pDCs, blue). (**B**) representative histograms showing the immunostaining of DRD1 in BDCs (left panel), mDCs (midle panel) and pDCs (right panel). Grey histograms represent fluorescence associated to isotype controls.

Figure S10., Prado et al.

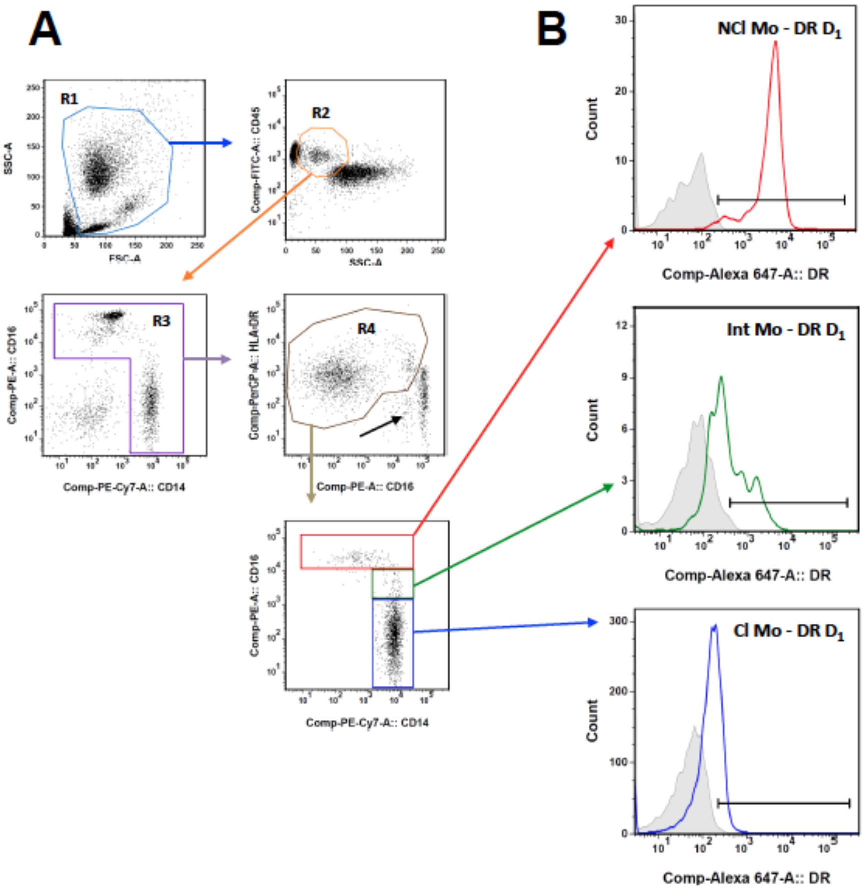

Figure S10. Gating strategy for the analysis of dopamine receptors in peripheral blood monocytes obtained from human individual. Fresh blood samples obtained from healthy donors were immunostained with antibodies specific to different DRs and to surface markers associated to monocytes (CD45, CD16, CD14 and HLA-DR) and different sub-populations of monocytes were analysed by flow cytometry. (**A**) Representative dot-plots showing the gating startegy used for selection of non-classical (NCl Mo, red), intermediate (Int Mo, green) and classical (Cl Mo, blue) monocytes. (**B**) representative histograms showing the immunostaining of DRD1 in NCl Mo (top panel), Int Mo (midle panel) and Cl Mo (bottom panel). Grey histograms represent fluorescence associated to isotype controls.

Figure S11., Prado et al.

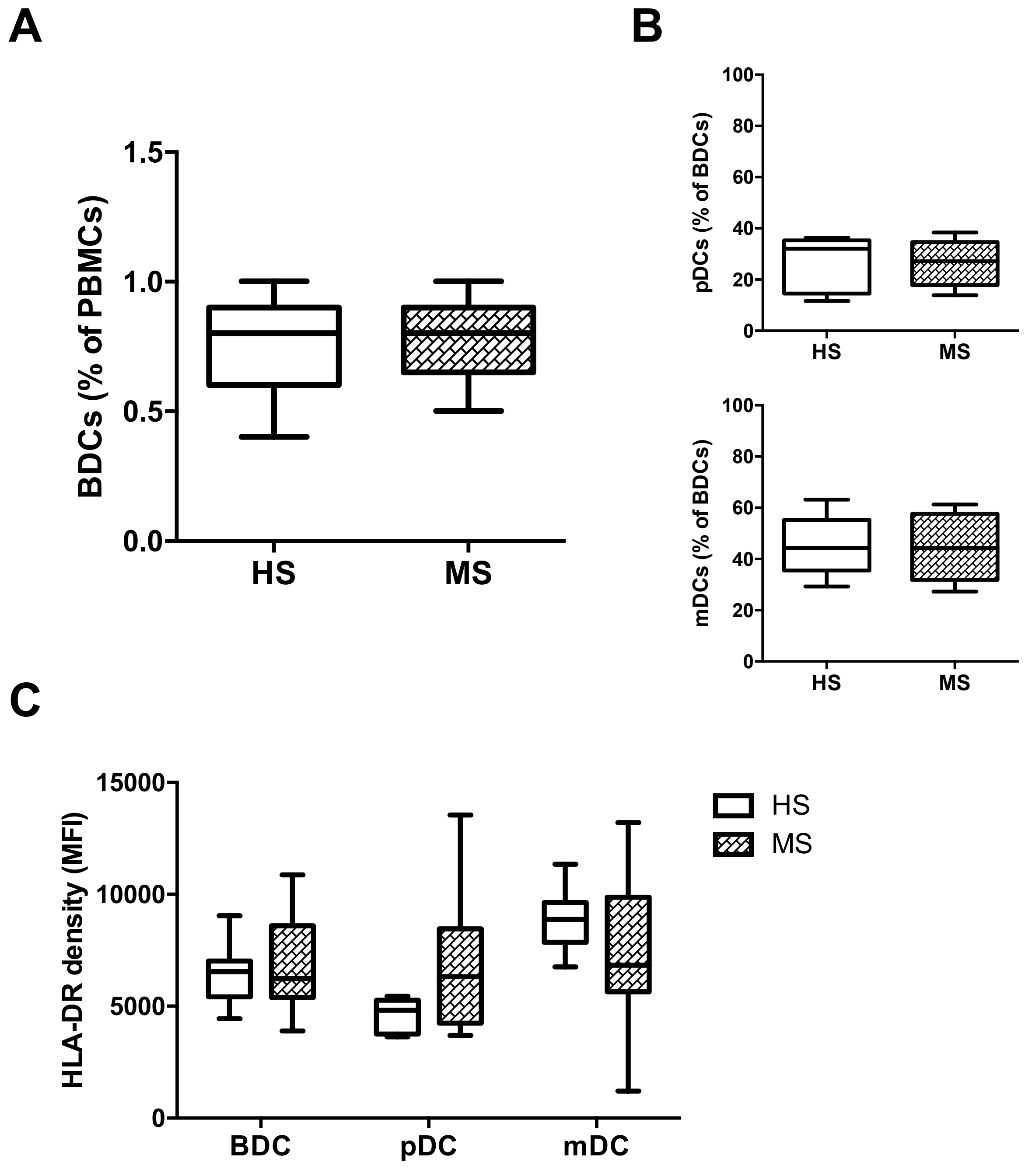

Figure S11. MS patients display unaltered frequencies of DCs and expression of HLA-DR. Fresh blood samples were obtained from healthy subjects (HS, n = 10; white bars) or multiple sclerosis patients (MS, n = 9; striped bars) and the frequencies of pDCs, mDCs and total BDCs and the expression of HLA-DR in these different DCs populations was analysed by flow cytometry using the gating strategy described in figure S9. Quantification of frequencies of total BDCs (**A**), pDCs (top panel) and mDCs (bottom panel) (**B**) are shown. The surface density of HLA-DR in BDCs, pDCs and mDCs was quantified as the MFI associated to the immunostaining for HLA-DR (**C**). (A-C) Values represent mean ± SEM. Not significant differences were found.

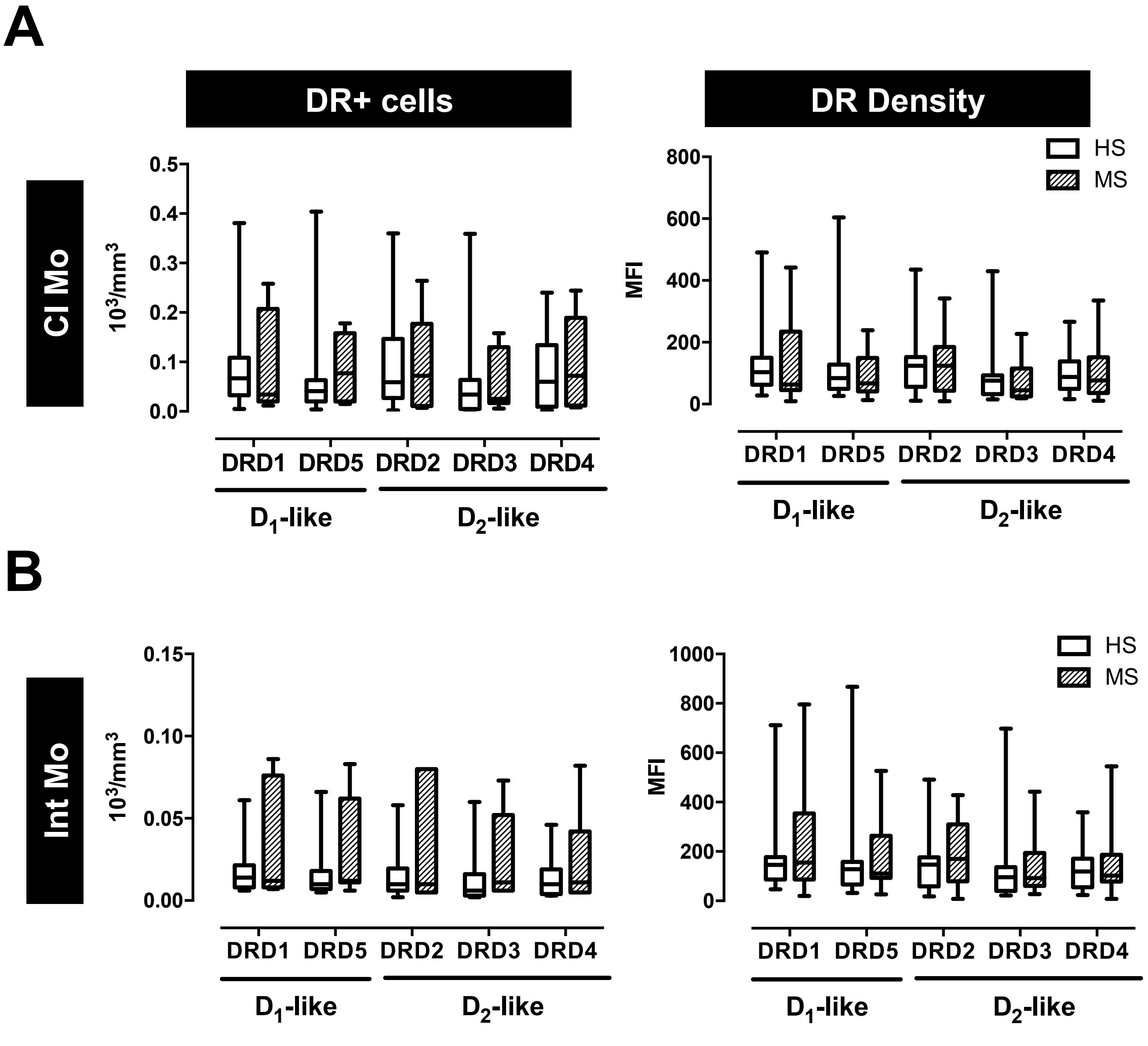

Figure S12. MS patients display unaltered expression of DRD5 in peripheral blood monocytes. Fresh blood samples were obtained from healthy subjects (HS, n = 10; white bars) or multiple sclerosis patients (MS, n = 9; striped bars) and the expression of DRs (D1-like and D2-like) was analysed in different monocyte populations as the number of cells expressing each receptors (left panels) or the MFI associated to the immunostaining of each receptor (right panels). The expression of DRs in classical monocytes, Cl Mo (**A**), and intermediate monocytes, Int Mo (**B**) were analysed based in the gating strategy described in figure supplementary 8. Values represent mean ± SEM. Not significant differences were found.

Not edited blots for Figure 2A

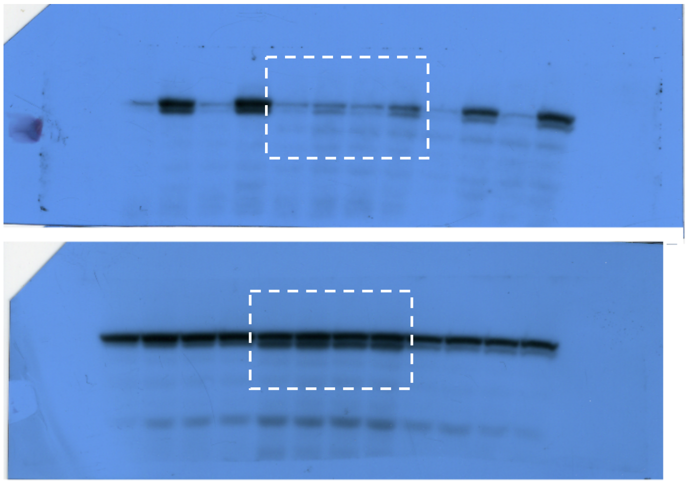

Not edited blots for Figure 3A - Top

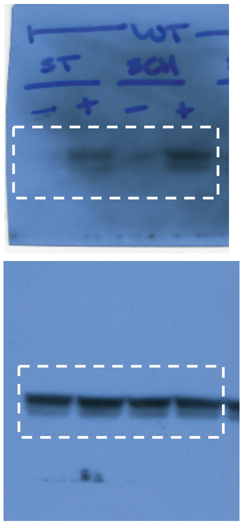

Not edited blots for Figure 3A - Bottom

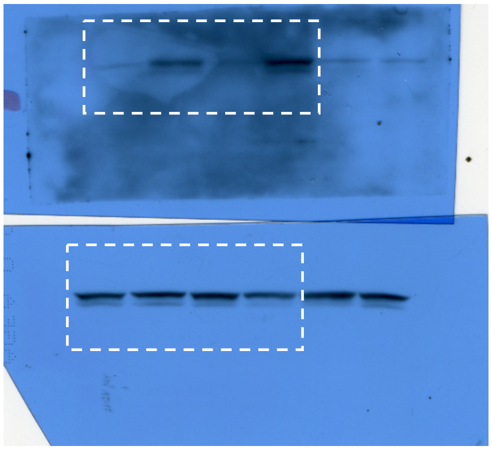

Figure S13. Not edited blots. Not edited blots from figures 2A and 3A are shown. Doted white lines show the place of the image that was used for edited figures 2A and 3A respectively.
